# Application research of image super-resolution reconstruction technology based on diffusion model in 3D digital image correlation

**DOI:** 10.1038/s41598-026-44638-7

**Published:** 2026-04-02

**Authors:** Dan Zhou, Hao Li, Chenxi Yao, Hao Chen

**Affiliations:** 1 Wenzhou University of Technology, Longwan Economic and Technological Development Zone, No. 337 Jinhai 3Rd Road, Wenzhou, 325000 China; 2 University of Macau, Macau, China; 3 Zhejiang University, Hangzhou, China

**Keywords:** Image super-resolution reconstruction, 3D digital image correlation, Diffusion model, Generative adversarial network, Single-camera stereo vision, Engineering, Mathematics and computing, Optics and photonics

## Abstract

3D digital image correlation (3D-DIC) is a pivotal non-contact full-field measurement method in experimental mechanics. Traditional 3D-DIC systems rely on multi-camera setups, which face challenges such as high hardware costs, complex synchronization, and limited stereo matching accuracy. Single-camera stereo vision mitigates synchronization issues but introduces inherent resolution loss, compromising measurement accuracy. To address this, we propose LaESR-Diff, a super-resolution diffusion model integrating an optimized enhanced super-resolution generative adversarial network (ESRGAN) with Laplacian noise scheduling. The LaESR-Diff model employs an optimized ESRGAN to generate conditional inputs, enhancing residual-in-residual dense blocks (RRDBs) and incorporating a zero-mean normalized cross-correlation (ZNCC) loss term for speckle image fidelity. A Laplacian noise schedule replaces traditional linear/cosine schedules to preserve high-frequency textures. A dual evaluation system combines general image metrics and 3D-DIC-specific accuracy metrics. Experiments on the “Stereo-DIC Challenge 1.0” dataset show LaESR-Diff achieves PSNR = 26.13 and SSIM = 0.7423 at × 8 scale, reducing surface height and displacement errors by 58.6% and 67.2%, respectively, compared to bicubic interpolation. Laboratory tensile tests confirm LaESR-Diff reduces full-field displacement error in a quadrangular-prism-based virtual stereo system (QVSS) from 4.25% to 1.71%, nearing the basic stereo system (BSS) accuracy. The results demonstrate that the proposed method effectively compensates for the accuracy degradation caused by resolution loss in single-camera systems. This study provides an effective solution for single-camera 3D-DIC accuracy enhancement, with potential applications in other image-based non-contact measurement fields.

## Introduction

Three-dimensional digital image correlation (3D-DIC) technology, as a crucial tool in non-contact full-field measurement, enables high-precision non-contact measurement of three-dimensional deformation by analysing the spatial displacement field of speckle patterns on object surfaces. This technology has been widely applied in key areas such as material mechanical property testing, structural health monitoring, and aerospace component inspection^1–5^. Traditional 3D-DIC systems rely on binocular or multi-view visual architectures, requiring at least two strictly synchronized cameras to capture images simultaneously from different perspectives for 3D deformation field reconstruction via stereo matching algorithms^6,7^.

However, in practical applications, the requirement for two synchronized cameras often makes conventional stereo DIC systems expensive and complex to construct, particularly when measuring transient deformations with high-speed or ultra-high-speed cameras^8^. In such cases, the need for two synchronized high-speed cameras not only significantly increases hardware costs but also introduces synchronization complexities^9,10^. Moreover, experimental constraints or physical camera limitations often restrict their applicability^9,11^. Additionally, nonlinear geometric distortions and intensity variations between image pairs captured by two cameras with different optical characteristics and orientations may inevitably lead to difficulties in achieving precise stereo matching, thereby reducing measurement accuracy^12^.

To overcome these limitations, researchers have proposed single-camera stereo vision methods based on optical assistance^2^. These approaches introduce optical elements into the camera’s light path, enabling a single camera to capture images from two or more virtual perspectives in a single shot. Representative works include:^10^ established a single-camera stereo vision system based on a biprism for 3D-DIC and validated its accuracy in 3D shape and deformation measurement through extensive experiments, laying the foundation for the application of single-camera stereo vision in 3D-DIC;^9^ combined a planar-mirror-assisted single-camera stereo vision system with DIC to successfully measure the out-of-plane deformation of an aluminium plate under shock wave impact, expanding the application scenarios of single-camera stereo vision 3D-DIC;^13^ developed a compact and portable single-camera stereo vision system for 3D-DIC using a triangular prism and two mirrors, further enhancing the portability and flexibility of the measurement system. Compared to traditional 3D-DIC techniques, single-camera stereo vision solutions demonstrate significant advantages: complete elimination of multi-camera synchronization timing errors; reduction of hardware costs by at least one camera; and more compact system designs. However, this approach also introduces new technical challenges—inherent resolution loss. A simple estimation reveals that in a single-camera stereo vision measurement system with *n* virtual perspectives; each virtual perspective loses approximately (*n* − 1)/*n* of the effective image resolution. This information loss directly leads to reduced deformation measurement accuracy, becoming a bottleneck limiting the performance of single-camera systems.

Image super-resolution (SR) reconstruction technology offers a new solution to this bottleneck^14,15^. Image SR reconstruction aims to restore or enhance low-resolution images into high-resolution images through algorithms, evolving from traditional methods to deep learning models^16^. Deep learning-based SR methods have surpassed traditional approaches in reconstruction performance^17–20^. From^21^, who first introduced convolutional neural networks (CNNs) to the field of image SR, to the widespread application of generative adversarial networks (GANs)^22–25^, which have become the mainstream framework in this field, and the recent rise of diffusion models^26–32^, image SR technology continues to advance, with reconstruction performance steadily improving^33,34^.

However, applying advanced image SR technology to the 3D-DIC field still faces unique challenges: First, existing SR research primarily focuses on natural images in public datasets, lacking specialized consideration for the characteristics of speckle images in DIC scenarios. Compared to natural images, speckle images in DIC scenarios contain more high-frequency and fine textures, making it difficult to achieve high-fidelity SR images using existing SR methods. Second, existing SR methods typically prioritize perceptual quality improvement when designing loss functions and evaluation metrics, often neglecting pixel-level misalignment. While the human eye is insensitive to minor pixel-level shifts, 3D-DIC scenarios demand extremely high measurement accuracy, rendering general SR models ineffective in preserving critical displacement and deformation information. Third, existing studies predominantly use general image quality evaluation metrics such as peak signal-to-noise ratio (PSNR) and structural similarity index (SSIM), lacking an evaluation system directly linked to DIC measurement accuracy. As a result, high scores on these metrics do not guarantee high accuracy in deformation measurement.

To address these research gaps, the core contributions of this study are as follows: First, to tackle the issue of reduced measurement accuracy due to insufficient resolution in single-camera 3D-DIC, a conditional diffusion model-based image SR framework is constructed. This framework achieves breakthroughs by optimizing noise scheduling strategies and conditional input mechanisms. On one hand, the loss function of the ESRGAN model is improved to better adapt to the features of speckle images in DIC scenarios, and the optimized ESRGAN model is used to generate conditional images for the diffusion model. On the other hand, the noise schedule of existing diffusion models is redesigned, adopting a Laplacian noise schedule to enhance reconstruction performance. Second, a dual evaluation system is proposed, incorporating both general image quality metrics and 3D-DIC-specific measurement accuracy metrics, to scientifically assess and validate the performance of image SR technology in 3D-DIC scenarios. Finally, systematic experimental validation confirms the superiority of the proposed image SR model: performance is verified on a public dataset in the 3D-DIC field for standard measurement scenarios, and laboratory tensile tests further validate the model’s performance in conventional and single-camera 3D-DIC scenarios.

## Related work

### Principles of 3D-DIC

**Fig. 1:**
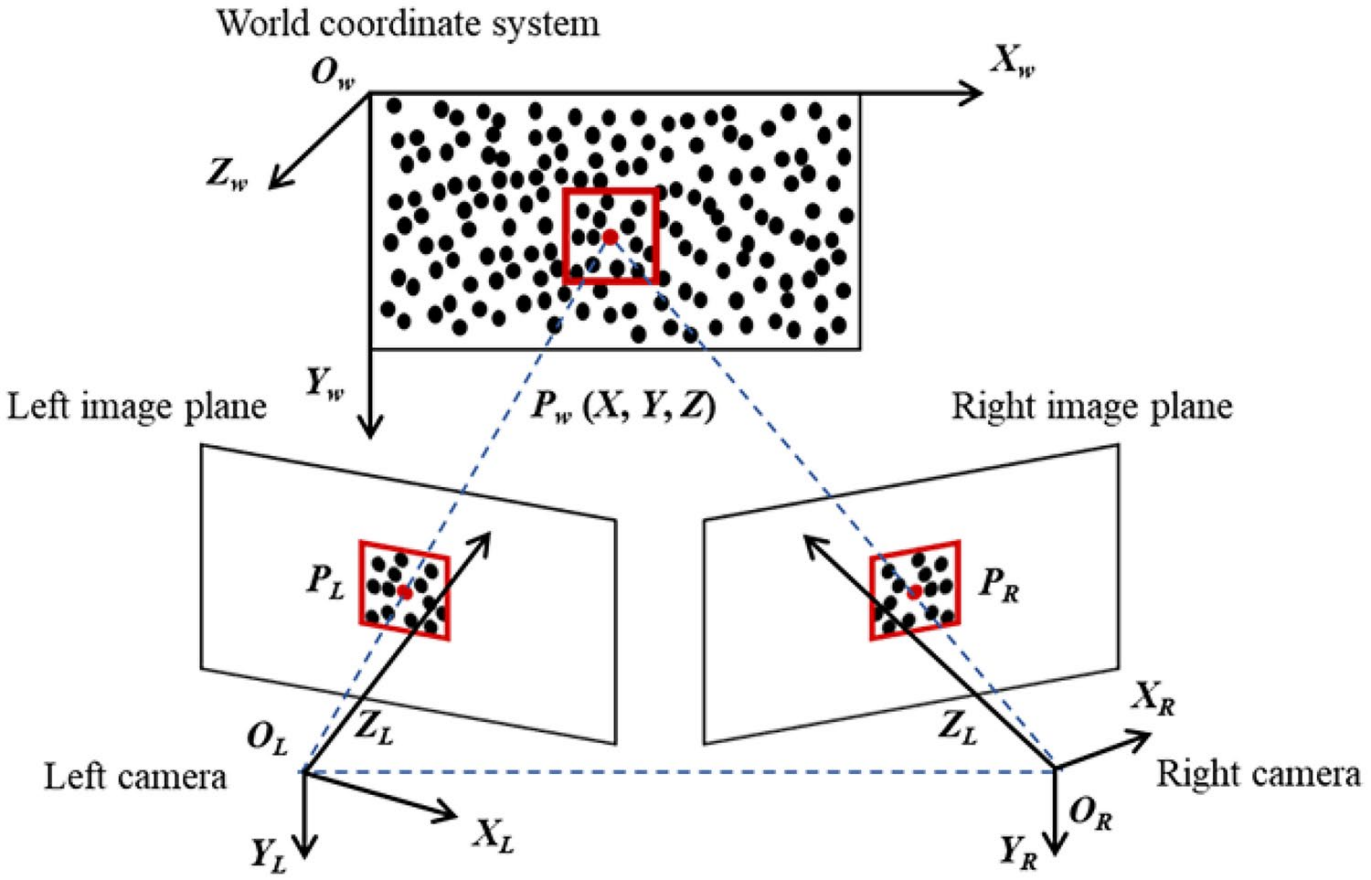
Basic principles of 3D-DIC.

3D-DIC is a three-dimensional deformation measurement method based on stereo vision and DIC principles^35,36^. As shown in Fig. 1, the measurement system uses two cameras to simultaneously record the deformation process of a specimen with a speckle pattern.

#### Stereo vision

In a stereo vision system, given the following prior conditions: 1) the intrinsic parameters (including effective focal length, principal point coordinates, and distortion coefficients) and extrinsic parameters (transformation matrix between the camera coordinate system and the world coordinate system) of the left and right cameras; 2) the extrinsic parameters between the two cameras (transformation matrix between the cameras); and 3) the projection coordinate pairs of a spatial point P on the left and right image planes, the three-dimensional coordinates of point P in the world coordinate system can be calculated according to stereo vision principles^37–39^. Here, 1) and 2) can be obtained through camera calibration, while 3) requires pixel matching.

#### Pixel matching

**Fig. 2:**
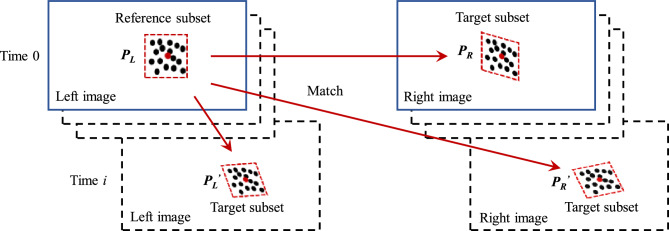
Pixel matching.

As shown in Fig. 2, pixel matching includes matching between left and right images and matching between images of the target at different times during deformation. The zero-mean normalized cross-correlation function (ZNCC) is typically used for matching^7,40^:

1 $$C_{ZNCC} = \frac{{\sum {\sum {[F(x,y) - \overline{F}]} } [G(x{\prime} ,y{\prime} ) - \overline{G}]}}{{\sqrt {\sum {\sum {[F(x,y) - \overline{F}]} }^{2} } \times \sqrt {\sum {\sum {[G(x{\prime} ,y{\prime} ) - \overline{G}]^{2} } } } }}$$

where $F(x,y)$ is the grayscale function of the reference image subset, G(*x*’,*y*’) is the grayscale function of the target image subset, and $\overline{F}$ and $\overline{G}$ represent the average grayscale of the reference and target image subsets, respectively. A higher ${C}_{ZNCC}$ indicates greater similarity between the reference and target subsets.

### Principles of single-camera stereo vision

Single-camera stereo vision typically consists of one camera and structurally designed optical elements, leveraging the principles of light reflection and refraction to capture images from multiple virtual perspectives in a single shot. The advantages of this design include: complete elimination of synchronization errors inherent in traditional stereo vision, making it highly suitable for dynamic object measurements; more compact measurement systems conducive to portable device development; and significant hardware cost savings by reducing the need for additional cameras and synchronization trigger devices^8,41^. This study focuses on two types of single-camera stereo vision systems.

#### Planar mirror-based single-camera stereo vision

**Fig. 3:**
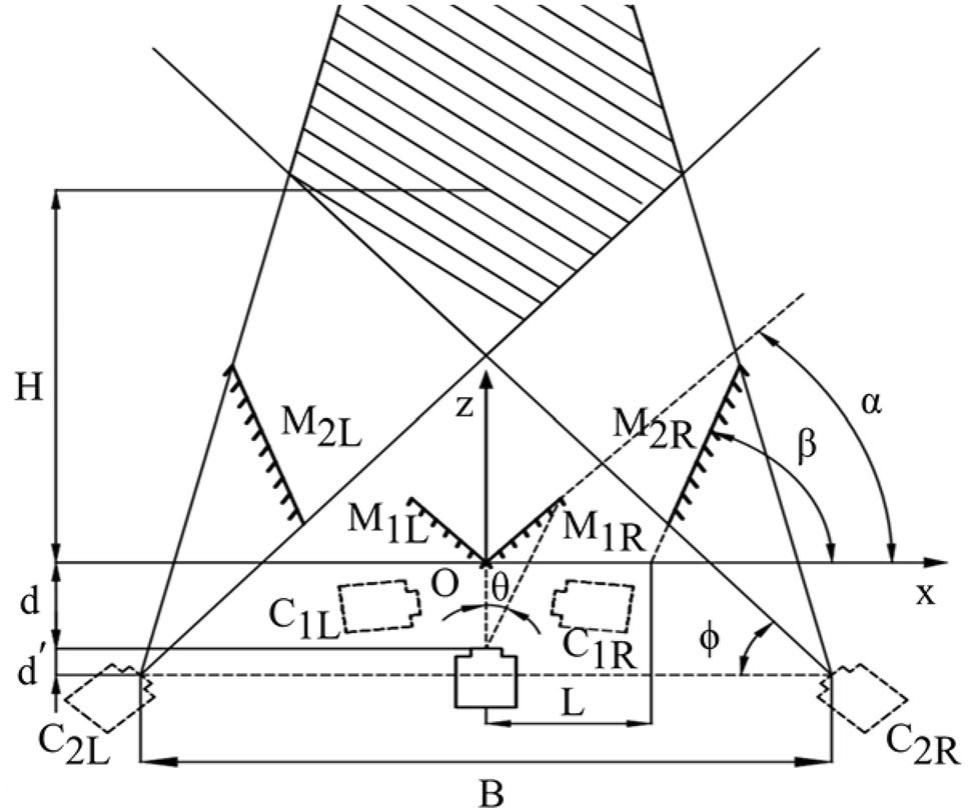
Geometric optical model of single camera stereo vision based on four planar mirrors.

The geometric optical model in Fig. 3 depicts a single-camera stereo vision system composed of a camera and four planar mirrors. Here, the four mirrors use optical reflection to direct light from the object to the same camera from two different perspectives. Specifically, the camera generates virtual cameras ${C}_{{1}L}$ and ${C}_{{1}R}$ through reflections from mirrors ${M}_{{1}L}$ and ${M}_{{1}R}$ , respectively. ${C}_{{1}L}$ and ${C}_{{1}R}$ further generate virtual cameras ${C}_{{2}L}$ and ${C}_{{2}R}$ via reflections from mirrors ${M}_{{2}L}$ and ${M}_{{2}R}$ , enabling stereo vision measurement by capturing two virtual perspectives in a single shot. Assuming a symmetric system where the intersection point *O* of mirrors ${M}_{{1}L}$ and ${M}_{{1}R}$ is the origin, the horizontal field of view of the real camera is the x-axis, and the depth direction is the z-axis, the relative positions of the camera and the four mirrors can be uniquely determined by four structural parameters: mirror placement angles *α* and *β*, the distance *d* between the real camera’s optical center and the origin, and the x-axis coordinate *L* of the intersection line of the mirrors. The placement angle $\phi$, baseline distance *B*, and field of view ranges ${\sigma }_{z}$ and ${\sigma }_{x}$ along the z- axis and x- axis of the virtual cameras ${C}_{{2}L}$ and ${C}_{{2}R}$ can be determined as follows^42^:

2 $$\phi ={90}^{\circ }+2\alpha -2\beta$$

3 $$B=2d\mathit{sin}2(\beta -\alpha )+4L{\mathit{sin}}^{2}\beta$$

4 $$\sigma_{Z} = \left\{ {\begin{array}{*{20}c} {H_{\min } = \frac{B}{2}\tan \phi - (d + d{\prime} )} \\ {H_{\max } = \frac{B}{2}\tan (\phi + \theta ) - (d + d{\prime} )} \\ \end{array} } \right.$$

5 $$\sigma_{x} = \left\{ {\begin{array}{*{20}c} {(H + d + d^{\prime})\cot \beta - \frac{B}{2}} & {\left[ {\frac{B}{2}\tan \phi \le H \le \frac{B}{\cot \phi + \cot (\phi + \theta )} - (d + d^{\prime})} \right]} \\ {\frac{B}{2} - (H + d + d^{\prime})\cot (\phi + \theta )} & {\left[ {H > \frac{B}{\cot \phi + \cot (\phi + \theta )} - (d + d^{\prime})} \right]} \\ \end{array} } \right.$$

where ${H}_{min}$ and ${H}_{max}$ represent the coordinate ranges of the effective field of view along the z-axis, $H$ describes the z-axis coordinate of the effective field of view, and *d’* is the distance between the virtual camera ${C}_{2L}$ (or ${C}_{2R}$) and the real camera along the z-axis.

#### Prism-based single-camera stereo vision

**Fig. 4:**
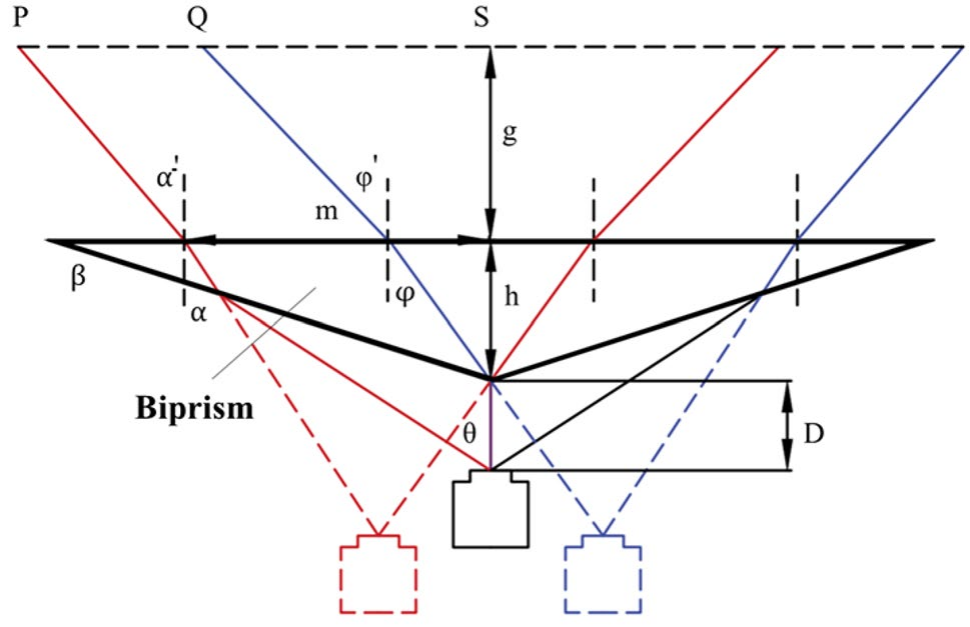
Geometric optical model of single camera stereo vision based on biprism.

Placing a prism in front of the camera lens allows the camera to capture images from different perspectives in a single shot due to light refraction. Fig. 4 illustrates the geometric optical model of a single-camera stereo vision system based on a biprism, where red lines represent the light path of the left virtual camera, blue lines represent the right virtual camera, and the thick line represents the biprism.

Assuming a symmetric system, the geometric relationships can be determined by three structural parameters: the base angle *β* and height *h* of the biprism, and the distance *D* between the camera’s optical center and the prism’s apex. If the real camera’s field of view angle $\theta$ is known, the effective field of view range can be determined using the angles α’ and φ’ between the light rays and the normal to the biprism’s bottom boundary. According to the law of refraction, the refraction behaviour at the biprism’s bottom and inclined edges can be described as:

6 $$\left\{ {\begin{array}{*{20}c} {\sin \alpha ^{\prime} = k\sin \alpha } \\ {\sin \varphi ^{\prime} = k\sin \varphi } \\ \end{array} } \right.$$

7 $$\left\{\begin{array}{c}\mathit{sin}\left(\theta +\beta \right)=k\mathit{sin}\left(\alpha +\beta \right)\\ \mathit{sin}\beta =k\mathit{sin}\left(\beta -\varphi \right)\end{array}\right.$$

Where *k* is the refractive index of the prism material. Solving these equations yields α’ and φ’. Further geometric derivations allow the calculation of the field of view width $PS$ and *QS* at a distance *g* from the biprism’s bottom edge:

8 $$\left\{ {\begin{array}{*{20}c} {PS = m + g\tan \alpha^{\prime}} \\ {QS = h\tan \varphi + g\tan \varphi^{\prime}} \\ {m = \frac{D\sin \theta (\cos \beta - \sin \beta \tan \alpha )}{{\sin (90 - \theta - \beta )}} + h\tan \alpha } \\ \end{array} } \right.$$

Additionally, changing the prism’s geometric shape, such as using a triangular or polygonal prism, can increase the number of virtual cameras. More complex geometric optical derivations can provide corresponding system designs, which are not discussed in detail here.

### Enhanced super-resolution generative adversarial network

Since SRGAN^22^ first introduced generative adversarial networks (GANs) to the field of image SR, GAN-based methods have gradually become a mainstream direction. The training process can be viewed as a Min–Max problem^25^, defined as:

9 $$\underset{{\theta }_{G}}{\mathit{min}}\underset{{\theta }_{D}}{\mathit{max}}{\mathbb{E}}_{{I}_{HR}\sim {p}_{train}\left({I}_{HR}\right)}\left[\mathit{log}{D}_{{\theta }_{G}}\left({I}_{HR}\right)\right]+{\mathbb{E}}_{{I}_{LR}\sim {p}_{train}\left({I}_{LR}\right)}\left[\mathit{log}\left(1-{D}_{{\theta }_{G}}\left({G}_{{\theta }_{G}}\left({I}_{LR}\right)\right)\right)\right]$$

where 𝔼 denotes the mathematical expectation, ${I}_{HR}\sim {p}_{train}\left({I}_{HR}\right)$ and ${I}_{LR}\sim {p}_{train}\left({I}_{LR}\right)$ represent high-resolution (HR) and low-resolution (LR) images sampled from the training set, respectively, ${D}_{{\theta }_{G}}$(·) is the discriminator network, and ${G}_{{\theta }_{G}}$(·) is the generator network.

ESRGAN^23^, an improved version of SRGAN, optimizes the generator architecture, discriminator design, and loss function. Its core architecture consists of three components:

#### Generator

The generator employs Residual-in-Residual Dense Blocks (RRDBs) as basic building units. Combining multi-level residual networks and dense connections, the output *y*_*i*_ of the *i*-th RRDB module for input *x*_*i*_ can be expressed as:

10 $$y_i=x_i\hspace{0.17em}+\hspace{0.17em}\alpha \cdot f_{RRDB}(x_i)$$

where *α* is the residual scaling factor, and *f*_*RRDB*_(·) represents the nonlinear transformation of the RRDB module. This structure enhances network capacity while residual connections alleviate gradient vanishing or explosion issues in deep networks, ensuring stable convergence.

#### Discriminator

A relativistic average discriminator is used to more accurately assess the difference between generated and real images. It estimates the probability that real data is more realistic than fake data on average, rather than simply predicting real or fake. This relativistic discriminator can be expressed as:

11 $$\left\{\begin{array}{c}{D}_{Ra}\left({I}_{r},{I}_{f}\right)=\sigma \left(C\left({I}_{r}\right)-{\mathbb{E}}_{{I}_{f}}\left(C\left({I}_{f}\right)\right)\right) \to 1 \left(more real than fake\right)\\ {D}_{Ra}\left({I}_{f},{I}_{r}\right)=\sigma \left(C\left({I}_{f}\right)-{\mathbb{E}}_{{I}_{r}}\left(C\left({I}_{r}\right)\right)\right) \to 0 \left(less real than real\right)\end{array}\right.$$

where ${\mathbb{E}}_{{I}_{f}}$ and ${E}_{{I}_{r}}$ denote the average operations over mini-batches of fake and real data, respectively. The discriminator loss function ${l}_{D}^{Ga}$ is defined as:

12 $${l}_{D}^{Ga}\mathrm{=}-{\mathbb{E}}_{{I}_{r}}\left[{\mathrm{log}}\left({D}_{Ra}\left({I}_{r},{I}_{f}\right)\right)\right]-{\mathbb{E}}_{{I}_{f}}\left[{\mathrm{log}}\left({{1}-D}_{Ra}\left({I}_{f},{I}_{r}\right)\right)\right]$$

where ${D}_{Ra}\left({I}_{r},{I}_{f}\right)$ represents the discriminator’s score for how real the real image ${I}_{r}$ is relative to the generated image ${I}_{f}$. This loss function drives the discriminator to more accurately evaluate the relative realism between images. Correspondingly, the generator adversarial loss ${l}_{G}^{Ga}$ is

13 $${l}_{G}^{Ga}\mathrm{=}-{\mathbb{E}}_{{I}_{r}}\left[{\mathrm{log}}\left({1}-{D}_{Ra}\left({I}_{r},{I}_{f}\right)\right)\right]-{\mathbb{E}}_{{I}_{f}}\left[{\mathrm{log}}\left({D}_{Ra}\left({I}_{f},{I}_{r}\right)\right)\right]$$

this adversarial training enables the generator to better utilize gradients from generated and real data, restoring more realistic textures. 

#### Loss function

 The total generator loss comprises perceptual loss ${l}_{percep}$, adversarial loss ${l}_{G}^{Ga}$, and content loss ${l}_{1}$. The perceptual loss ${l}_{percep}$ typically uses features extracted from a pre-trained VGG network (e.g., VGG16’s third or fifth layer). The content loss ${l}_{1}$ is the L1-norm loss. The total loss function ${l}_{ESRGAN}$ is:

14 $${l}_{ESRGAN}\mathrm{=}{l}_{percep}\mathrm{+}\lambda {l}_{G}^{Ga}\mathrm{+}\eta {l}_{1}$$

where $\lambda$ and $\eta$ are balancing coefficients to adjust the relative importance of different loss terms.

### Diffusion model-based image super-resolution

**Fig. 5:**
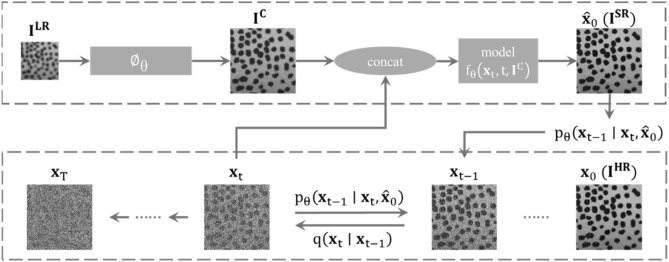
Schematic diagram of the overall framework of diffusion model.

The basic principles of diffusion models include two stages: forward diffusion and reverse diffusion^31^. The overall architecture is shown in Fig. 5. In the forward diffusion stage, the model gradually injects noise into the original high-resolution image until it becomes pure noise. The reverse diffusion stage reverses this process, starting from the noisy image and gradually denoising to restore high-resolution details. Given an image SR dataset $({I}^{HR},{I}^{LR})$, the diffusion model includes a series of diffusion variables $x = \left\{ {x_{t} |t = 0,1, \ldots ,T} \right\}$, where ${x}_{0}={I}^{HR}$ and ${x}_{T}$ follows a Gaussian distribution $\mathcal{N}\mathrm{(0,}\mathbf{I}\mathrm{)}$. The ultimate goal of the diffusion model is to map Gaussian noise ${x}_{T}\text{ }\mathcal{N}\mathrm{(0,}\mathbf{I}\mathrm{)}$ to an HR image ${I}^{LR}$ with the corresponding conditional image ${I}^{C}$, where ${I}^{C}$ is typically ${I}^{C}={I}^{LR}$ or ${I}^{C}\mathrm{=}{\varnothing }_{\theta }\mathrm{(}{I}^{LR}\mathrm{)}$, with ${\varnothing }_{\theta }$  being a pre-trained image SR model (see Sect. "Conditional Image Input" for details).

#### Forward diffusion

This process simulates the gradual degradation of an image from a clear state to pure noise. The stepwise addition of noise is controlled by a carefully designed noise schedule, defined by coefficient pairs $\{\alpha , \sigma \}=\{{\alpha }_{t}, {\sigma }_{t}|t=\mathrm{0,1},\dots \dots ,T\}$. The mathematical description can be formalized using a Markov chain $q$:

15 $$q({x}_{t}\mid {x}_{t-{1}})\mathrm{=}\mathcal{N}({x}_{t}\mathrm{;}\frac{{\alpha }_{t}}{{\alpha }_{t-{1}}}{x}_{t-{1}}\mathrm{,(1-}\frac{{\lambda }_{t}}{{\lambda }_{t-{1}}}\mathrm{)}{\sigma }_{t}^{2}\mathbf{I})$$

where $\mathcal{N}$ denotes a normal distribution, $\mathbf{I}$ is the identity matrix, and ${\lambda }_{t}\mathrm{=}{\alpha }_{t}^{2}/{\sigma }_{t}^{2}$ decreases as the time step *t* increases. Leveraging the properties of Gaussian distributions, the intermediate steps can be marginalized to express the relationship between ${x}_{t}$ and ${x}_{0}$:

16 $$q({x}_{t}\mid {x}_{0})\mathrm{=}\mathcal{N}({x}_{t}\mathrm{;}{\alpha }_{t}{x}_{0}\mathrm{,}{\sigma }_{t}^{2}\mathbf{I})$$

Further, ${x}_{t}$ can be expressed as:

17 $${x}_{t}\mathrm{=}{\alpha }_{t}\cdot {x}_{0}\mathrm{+}{\sigma }_{t}\cdot \epsilon$$

where $\epsilon$ is a Gaussian noise vector satisfying $\epsilon \sim \mathcal{N}(0\mathrm{,}\mathbf{I})$. The model is trained to obtain a network ${f}_{\theta }$ such that ${{\widehat{x}}_{0}\mathrm{=}f}_{\theta }\left({x}_{t}\mathrm{,}t\mathrm{,}{I}^{C}\right)\approx {x}_{0}$. The loss function $L$ is defined as:

18 $${L\mathrm{=}{\mathbb{E}}}_{t\mathrm{,}{(x}_{0}\mathrm{,}{I}^{C}\mathrm{),}\epsilon }{\Vert {f}_{\theta }\left({\alpha }_{t}\cdot {x}_{0}\mathrm{+}{\sigma }_{t}\cdot \epsilon \mathrm{,}t\mathrm{,}{I}^{C}\right)-{x}_{0}\Vert }_{m}^{m}$$

where $\epsilon \sim \mathcal{N}(0\mathrm{,}\mathbf{I})$ and $m\in \mathrm{[1,2]}$. The training pseudo-code is shown in Table 1.

**Table 1:** Training pseudo code.

Training
Input: Dataset *D*, timesteps *T*, noise schedule$\{\alpha , \sigma \}$**,** pre-trained SR model${\varnothing }_{\theta }$,* m* = 1 or 2
1: **repeat**
2: ($${I}^{HR},{I}^{LR}$$)$$\sim D$$, *t*$$\sim \mathrm{Uniform}(\{\mathrm{1,2},\dots \dots ,T\})$$,$$\epsilon \sim \mathcal{N}(0,\mathbf{I})$$
3:$${{\boldsymbol{x}}}_{0}={I}^{HR}$$,$${I}^{C}={\varnothing }_{\theta }({I}^{LR})$$
4: Take a gradient descent step on
$${{\nabla }_{\theta }\Vert {f}_{\theta }\left({\alpha }_{t}\cdot {{\boldsymbol{x}}}_{0}+{\sigma }_{t}\cdot \epsilon ,t,{I}^{C}\right)-{{\boldsymbol{x}}}_{0}\Vert }_{m}^{m}$$
5: **until** converged

#### Reverse diffusion

To recover the original image x0 × 0 from the noisy image xT*xT*, the forward diffusion process must be reversed. This can be represented by a parameterized Markov chain ${p}_{\theta }$:

19 $${p}_{\theta }\left({x}_{t-{1}}|{x}_{t}\mathrm{,}{\widehat{x}}_{0}\right)\mathrm{=}\mathcal{N}\left({\alpha }_{t-{1}}{\widehat{x}}_{\theta }\left({x}_{t}\mathrm{,}t\right)\mathrm{,}{\sigma }_{t-1}^{2}\mathbf{I}\right)$$

where ${\widehat{x}}_{\theta }\left({x}_{t}\mathrm{,}t\right)$ is the predicted image at time *t*, obtained from ${f}_{\theta }\left({x}_{t}\mathrm{,}t\mathrm{,}{I}^{C}\right)$, and the noise ${z}_{t}$ can be predicted as ${z}_{t}\mathrm{=(}{x}_{t}-{\alpha }_{t}{\widehat{x}}_{0}\mathrm{)/}{\sigma }_{t}$. After training, given the starting point ${x}_{T}$, the original image ${x}_{0}$ can be iteratively reconstructed:

20 $$x_{t - 1} \leftarrow \alpha_{t - 1} f_{\theta } (x_{t} ,t,I^{C} ) + \frac{{\sigma_{t - 1} }}{{\sigma_{t} }}(x_{t} - \alpha_{t} f_{\theta } (x_{t} ,t,I^{C} ))$$

This iterative process is also called inference. The inference pseudo-code is shown in Table 2.

**Table 2:** Inference pseudo code.

Inference
Input: trained model ${f}_{\theta }$, pre-trained SR model ${\varnothing }_{\theta }$
1:$${x}_{T}\sim \mathcal{N}\left(0,\mathbf{I}\right)$$
2:$${I}^{C}={\varnothing }_{\theta }({I}^{LR})$$
3: **for** *t* = *T*,*T*-1,……,1 **do**
$$\epsilon \sim \mathcal{N}(0,\mathbf{I})$$, **if** *t* > 1, **else** $$\epsilon =0$$
$${x}_{t-1}={\alpha }_{t-1}{f}_{\theta }\left({x}_{t},t,{I}^{C}\right)+\frac{{\sigma }_{t-1}}{{\sigma }_{t}}\left({x}_{t}-{\alpha }_{t}{f}_{\theta }\left({x}_{t},t,{I}^{C}\right)\right)$$
4: **end for**
5: **return** $${x}_{0}$$

## Method

### Conditional image input

In diffusion model-based image SR tasks, the quality of conditional image inputs is decisive for the final reconstruction performance. Although using ESRGAN can produce high-quality conditional images, its loss function (including perceptual loss, generator loss, and L1-norm loss) tends to prioritize perceptual quality at the expense of pixel-level misalignment, affecting 3D-DIC measurement accuracy.

**Fig. 6:**
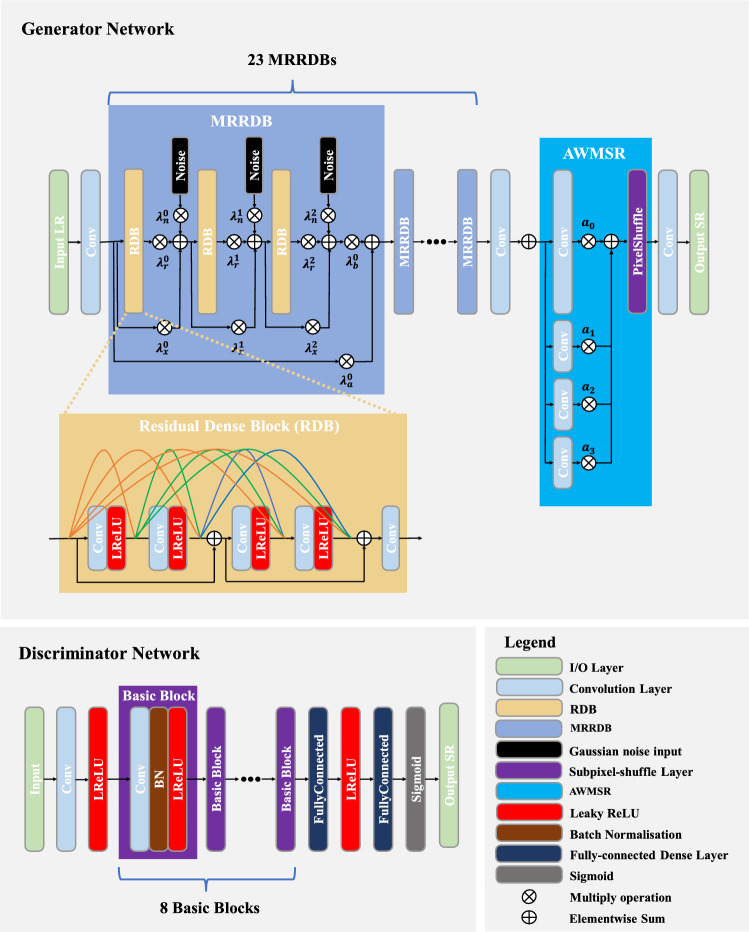
Optimized ESRGAN framework.

To address the specific requirements of 3D-DIC applications, this study optimizes the ESRGAN model with two main improvements:

1) Architecture Enhancement: Building on RRDB units, each dense block (DB) uses independent weights to improve information and gradient transfer efficiency. Inspired by the Adaptive Weight Multi-Scale Reconstruction (AWMSR) module, nonlinear mapping features are leveraged to achieve high-resolution outputs. The optimized model framework is shown in Fig. 6.

2) Loss Function Adjustment: A ZNCC-based loss term is added to align the optimization objective with 3D-DCI scenarios, ensuring better preservation of local structural consistency critical for displacement and deformation measurements.

The generator starts with a single convolutional layer (3 × 3 kernel, 64 feature maps, stride 1) for initial feature extraction:

21 $${x}_{0}\mathrm{=}{f}_{0}\left({I}_{LR}\right)$$

where ${f}_{0}$ is the initial feature extraction function for the LR image ${I}_{LR}$, and ${x}_{0}$ is the output feature map. The generator employs multi-adaptive-weights residual-in-residual dense blocks (MRRDBs), an improved version of ESRGAN’s RRDB units^43^. Unlike ESRGAN’s fixed residual scaling of 0.2, this study uses independent weights for each DB, allowing adaptive learning to enhance information and gradient flow. For the *n*-th MRRDB unit, the input ${x}_{n}$ and output ${x}_{n+{1}}$ are:

22 $${x}_{n+{1}}\mathrm{=}{f}_{MRRDB}\left({x}_{n}\right)\mathrm{=}{\lambda }_{b}^{n}\left({x}_{{n}_{6}}\right)\mathrm{+}{\lambda }_{a}^{n}{x}_{n}$$

where ${\lambda }_{a}^{n}$ and ${\lambda }_{b}^{n}$ are independent weights for the *n*-th MRRDB unit, and ${x}_{{n}_{6}}$ is derived from:

23 $$\left\{\begin{array}{c}{x}_{{n}_{1}}={\lambda }_{r}^{{n}_{0}}{f}_{RRDB}\left({x}_{n}\right)+{\lambda }_{x}^{{n}_{0}}{x}_{n}+{\lambda }_{n}^{{n}_{0}}{G}_{n}\\ {x}_{{n}_{2}}={\lambda }_{r}^{{n}_{1}}{f}_{RRDB}\left({x}_{{n}_{1}}\right)+{\lambda }_{x}^{{n}_{1}}{x}_{{n}_{1}}+{\lambda }_{n}^{{n}_{1}}{G}_{n}\\ {x}_{{n}_{3}}={\lambda }_{r}^{{n}_{2}}{f}_{RRDB}\left({x}_{{n}_{2}}\right)+{\lambda }_{x}^{{n}_{2}}{x}_{{n}_{2}}+{\lambda }_{n}^{{n}_{2}}{G}_{n}\\ {x}_{{n}_{4}}={\lambda }_{r}^{{n}_{3}}{f}_{RRDB}\left({x}_{{n}_{3}}\right)+{\lambda }_{x}^{{n}_{3}}{x}_{{n}_{3}}+{\lambda }_{n}^{{n}_{3}}{G}_{n}\\ {x}_{{n}_{5}}={\lambda }_{r}^{{n}_{4}}{f}_{RRDB}\left({x}_{{n}_{4}}\right)+{\lambda }_{x}^{{n}_{4}}{x}_{{n}_{4}}+{\lambda }_{n}^{{n}_{4}}{G}_{n}\\ {x}_{{n}_{6}}={\lambda }_{r}^{{n}_{5}}{f}_{RRDB}\left({x}_{{n}_{5}}\right)+{\lambda }_{x}^{{n}_{5}}{x}_{{n}_{5}}+{\lambda }_{n}^{{n}_{5}}{G}_{n}\end{array}\right.$$

where ${\lambda }_{r}^{{n}_{k}}$, ${\lambda }_{x}^{{n}_{k}}$, and ${\lambda }_{n}^{{n}_{k}}$ are three sets of independent weights for the RRDB unit, and ${G}_{n}$ is the input Gaussian noise. To better utilize the features of the nonlinear mapping module, the adaptive weight multi-scale reconstruction module^44^ (AWMSR) is adopted. At this time, the output ${x}_{n+{1}}$ will be used as the input of the AWMSR unit, and the SR image ${I}_{SR}$ can be represented as:

24 $${I}_{SR}\mathrm{=}{f}_{rec}\left(\sum_{i= \mathrm{0} }^{n}{\alpha }_{i}{f}_{AWMSR}^{i}\left({x}_{n+{1}}\right)\right)$$

where ${f}_{rec}$ is the final convolutional layer, ${f}_{AWMSR}^{i}$ is the *i*-th scale branch of the AWMSR module, and ${\alpha }_{i}$ is the adaptive weight for the *i* -th branch.

In terms of loss function design, this study introduces a loss term based on ZNCC. Firstly, define the average ZNCC:

25 $${A}_{ZNCC}(F,G,{A}_{K})=\frac{1}{K}\sum_{K=1}^{K}\frac{{\sum }_{\left(x,y\right)\in {A}_{K}}\left[F\left(x,y\right)-\overline{F}\right]\left[G\left({x}{\prime},{y}{\prime}\right)-\overline{G}\right]}{\sqrt{{\sum }_{\left(x,y\right)\in {A}_{K}}\sum {\left[F(x,y)-\overline{F}\right]}^{2}}\times \sqrt{{\sum }_{\left(x,y\right)\in {A}_{K}}{\left[G\left({x}{\prime},{y}{\prime}\right)-\overline{G}\right]}^{2}}}$$

where ${A}_{K}$ is a local patch of size *n* × *n* slid with stride *s* over images *F* and *G*. The ZNCC loss is:

26 $${l}_{ZNCC}=1-{A}_{ZNCC}({I}_{SR},{I}_{LR},{A}_{K})$$

The total loss function is:

27 $$l={l}_{ZNCC}+{l}_{ESRGAN}$$

The most significant advantage of designing the loss function in this way is that, since ZNCC is the most commonly used pixel matching index in 3D-DIC scenarios and plays a decisive role in the final displacement and deformation measurement, adding a loss term based on ZNCC can help maintain the consistency of the local image structure, which is suitable for the calculation requirements of 3D-DIC.

### Noise schedule

In diffusion models, the noise schedule, as the core parameter controlling the dynamic adjustment of noise in the forward and reverse diffusion processes, directly determines the generation performance and reconstruction accuracy of the model^31^. The initial diffusion models generally adopted a linear noise schedule, but this strategy has significant drawbacks: the noise decay rate in the early stage of the forward diffusion stage is too fast, resulting in the feature information of low-frequency structures (such as object contours) being diluted too early; in the later stage, the excessive noise redundancy makes it difficult to effectively separate high-frequency details (such as texture features). This limitation stems from the homogeneous treatment of features of different frequencies by the linear schedule—there are essential differences in the response mechanisms of low-frequency structures and high-frequency details to noise intensity.

In response to the above problems, researchers have proposed a variety of improved noise schedules (Table 3). Among them, the cosine noise schedule has become the current mainstream solution due to its stable noise decay characteristics^45–47^. However, the smoothness assumption of this method may lead to insufficient recovery of high-frequency details in complex texture areas, especially in the reverse diffusion stage, where excessive smoothing is likely to cause blurring of key features. In contrast, the probability density function of the Laplace distribution has a sharp peak and thick tail characteristics, which enables it to more sensitively capture sparse but key high-frequency information in images during the noise injection process. For example, in the super-resolution (SR) task of speckle images in the digital image correlation (DIC) scenario of this study, the Laplace noise schedule can effectively suppress the smoothing effect of the diffusion process on fine textures and retain the local gradient features of the speckle patterns.

**Table 3:** Noise schedule commonly used in diffusion models^48^.

Noise schedule	$\lambda (t)=\mathit{ln}\left(\frac{{\alpha }_{t}^{2}}{{\sigma }_{t}^{2}}\right)$
Cosine	$2\mathit{ln}\left[cot\left(\frac{\pi }{2}\cdot \frac{t}{T}\right)\right]$
Laplace{$\mu , b$}	$\mu -b sgn\left(0.5-\frac{t}{T}\right)\mathit{ln}\left(1-2\left|\frac{t}{T}-0.5\right|\right)$
Cauchy{$\mu$,$\gamma$}	$\mu +\gamma \mathit{tan}\left[\frac{\pi }{2}\left(1-\frac{2t}{T}\right)\right]$
Cosine_shifted{$\mu$}	$\mu +2\mathit{ln}\left[cot\left(\frac{\pi }{2}\cdot \frac{t}{T}\right)\right]$
Cosine_scaled{s}	$\frac{2}{s}\mathit{ln}\left[cot\left(\frac{\pi }{2}\cdot \frac{t}{T}\right)\right]$
$\{{\alpha }_{t}, {\sigma }_{t}$} can be calculated by ${\alpha }_{t}^{2}=\frac{{e}^{\lambda }}{{e}^{\lambda }+1}, {\sigma }_{t}^{2}=\frac{1}{{e}^{\lambda }+1}$	

Inspired by the research results of reference^48^, this study intends to use the Laplace noise schedule to replace the cosine noise schedule. This reference has verified the effectiveness and superiority of the Laplace noise schedule through the ImageNet benchmark test set. This study extends the application of the Laplace noise schedule to the field of image SR reconstruction, specifically, it further applies it to the SR reconstruction task of speckle images in the DIC scenario.

### Validation and evaluation methods

This study adopts four general image quality metrics in the field of image SR: 1) Peak Signal-to-Noise Ratio (PSNR); 2) Structural Similarity Index (SSIM)^49^; 3) Learned Perceptual Image Patch Similarity (LPIPS)^50^; 4) Natural Image Quality Evaluator (NIQE)^51^. These metrics provide a quantitative basis for the SR reconstruction quality from different dimensions: PSNR measures the pixel-level reconstruction accuracy, SSIM evaluates the structural similarity, LPIPS simulates the perceptual quality, and NIQE assesses the naturalness and distortion degree of images.

However, it should be particularly emphasized that the above metrics have obvious limitations. They are mainly used to evaluate the performance of SR models on common SR public datasets (such as DIV2K, Set5, etc.), and their optimization goals are essentially different from the 3D-DIC application scenarios focused on in this study. In the 3D-DIC scenario, the ultimate value of image SR reconstruction is not simply the improvement of visual quality, but whether it can effectively improve the final measurement accuracy of the system. Whether the general image quality metrics can accurately reflect the impact of image SR on the measurement performance of 3D-DIC remains to be verified by experiments.

In view of this, this study constructs a dual evaluation system: on the basis of retaining general image quality metrics, key measurement accuracy evaluation metrics specific to 3D-DIC are mainly introduced. Specifically, they include: 1) surface height reconstruction error; 2) displacement measurement accuracy; 3) deformation measurement accuracy and other key performance parameters (details are shown in Sect. "Public Dataset Experiment" and Sect. "Laboratory Experiment"). The advantage of this evaluation strategy is that it not only examines the image quality of SR reconstruction, but also focuses on verifying the actual gain of SR processing on 3D-DIC measurement. At the same time, it can further investigate the internal relationship between common SR evaluation metrics and measurement accuracy metrics, providing a more persuasive evaluation basis for the application of image SR technology in experimental mechanics.

## Public dataset experiment

### Dataset source

In this experiment, the public dataset used is from "Stereo-DIC Challenge 1.0"^52^, a challenge activity initiated by the International DIC Society (iDICs) to promote the further development of 3D-DIC technology. This challenge project provides a set of specimen image data captured in a strictly controlled experimental environment for in-depth analysis and research by scientific researchers and developers. It is worth mentioning that there was previously a lack of reference resources for benchmark testing and comparing the performance of current 3D-DIC algorithms in the industry^53^, and the release of this dataset just fills this gap, enabling researchers to test and improve algorithms on a unified and strictly controlled experimental basis. Therefore, this experiment also uses this dataset to verify the effectiveness of the image SR model in the 3D-DIC measurement scenario in a low-cost, high-efficiency, and benchmark-referenced manner.

**Fig. 7:**
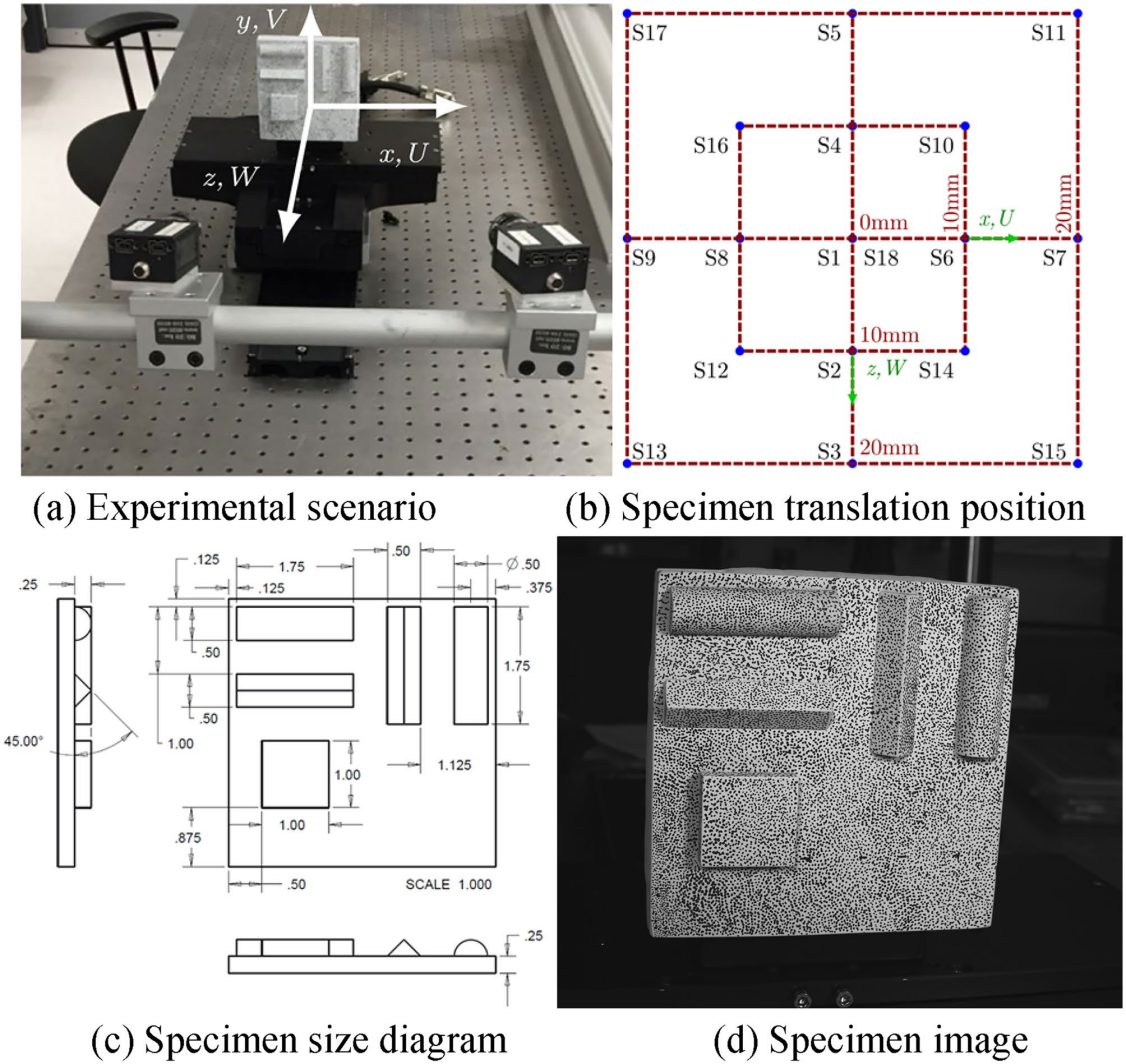
Overview of experimental settings^52^.

Fig. 7(a) is a schematic diagram of the laboratory scene for obtaining the dataset; Fig. 7(b) is a schematic diagram of the specimen’s movement, where S1—S18 represent the specimen in different positions; Fig. 7(c) is the design drawing of the specimen (the dimension units in the annotation are inches); Fig. 7(d) is an example of the images in the dataset. The dimensions of the specimen were measured by laser scanning, and the displacements were measured by a coordinate measuring machine for comparison with the results of 3D-DIC.

### Results and analysis

**Fig. 8:**
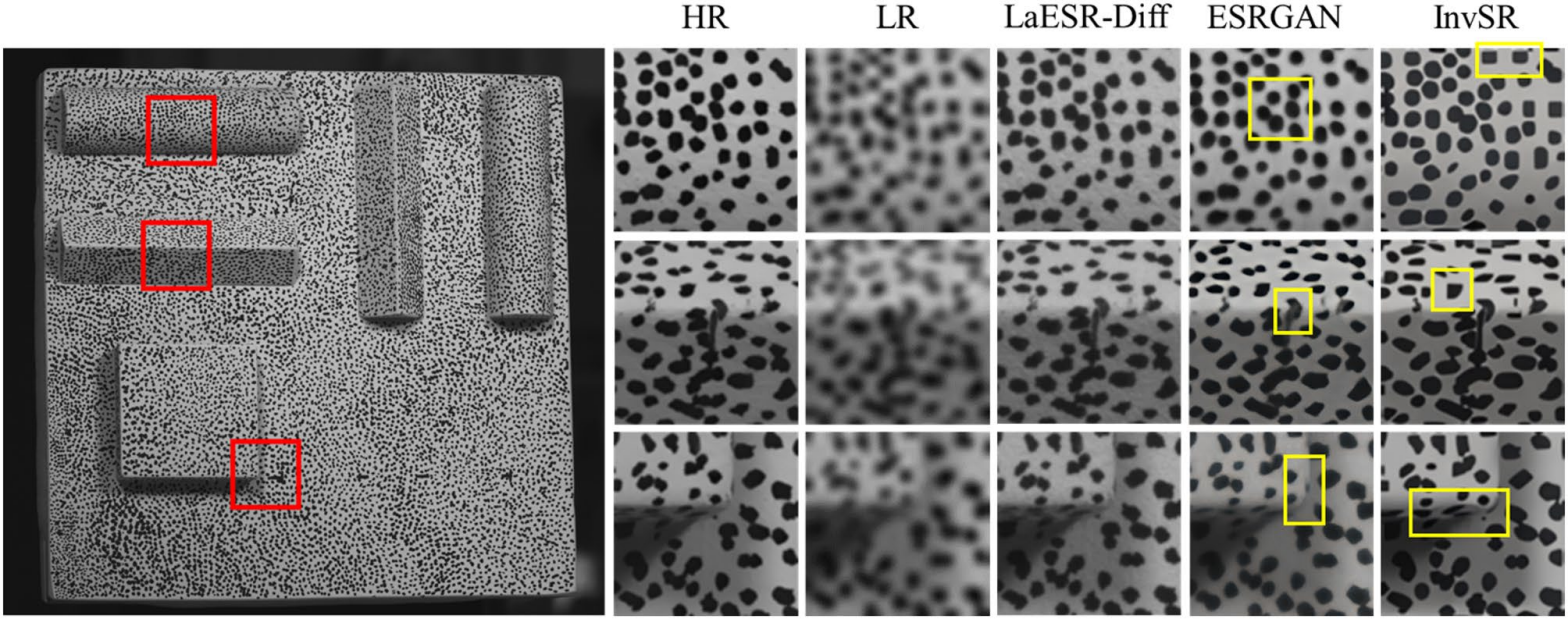
Qualitative comparison of image SR models on specimen image.

Fig. 8 presents a qualitative comparison of the local details of LR images after SR reconstruction using different methods. It can be seen that ESRGAN^19^, InvSR^28^, and the method of this study can all improve the visual effects of LR images to a certain extent. The yellow box lines indicate obvious distortion or misalignment in the local area after SR reconstruction.

Table 4 shows the quantitative results of the general image quality metrics for each SR model. The boxed marks represent the best values in a single column, and the underlines represent the second-best values in a single column. At different SR scales, LaESR-Diff, ESRGAN, and InvSR generally outperform Bicubic. As a traditional interpolation method, Bicubic estimates new pixel values by weighted averaging of neighboring pixels, without learning the complex features and structural information of the image, and it is a basic image super-resolution method. Therefore, it has limited ability in handling complex image details and texture restoration, and as the SR scale increases, the image quality evaluation metrics decrease significantly. Regarding the differences in metrics performance, LaESR-Diff has more advantages in PSNR and SSIM, while ESRGAN and InvSR perform better in LPIPS and NIQE. The reason is that LaESR-Diff may focus on optimizing the accurate prediction of image pixel values and the restoration of structural similarity in its model architecture and loss function design; ESRGAN optimizes the image perception effect through adversarial training and specific perceptual loss, and as a diffusion model, InvSR learns the image data distribution through a step-by-step denoising process, which can accurately restore high-frequency details and textures and better handle image perception features, so it performs well in LPIPS and NIQE.

**Table 4:** Quantitative results of general image quality evaluation metrics for SR model.

Model	PSNR ↑ /SSIM ↑ /LPIPS ↓ /NIQE ↓		
	× 2	× 4	× 8
Bicubic	31.55 / 0.8002 / 0.3991 / 5.784	29.19 / 0.7504 / 0.4870 / 7.017	21.36 / 0.5812 / 0.5917 / 7.489
**LaESR-Diff**	37.88 / 0.8964 / 0.2669 / 3.784	35.09 / 0.8522 / 0.3894 / 5.001	26.13 / 0.7423 / 0.3567 / 6.281
ESRGAN	37.52 / 0.8864 / 0.2591 / 4.243	34. 53 / 0.8642 / 0.2770 / 4.873	25.74 / 0.7064 / 0.4352 / 6.578
InvSR	37.01 / 0.9027 / 0.2741 / 3.481	34.34 / 0.8369 / 0.2879 / 4.245	26.09 / 0.7269 / 0.2937 / 5.381

**Fig. 9:**
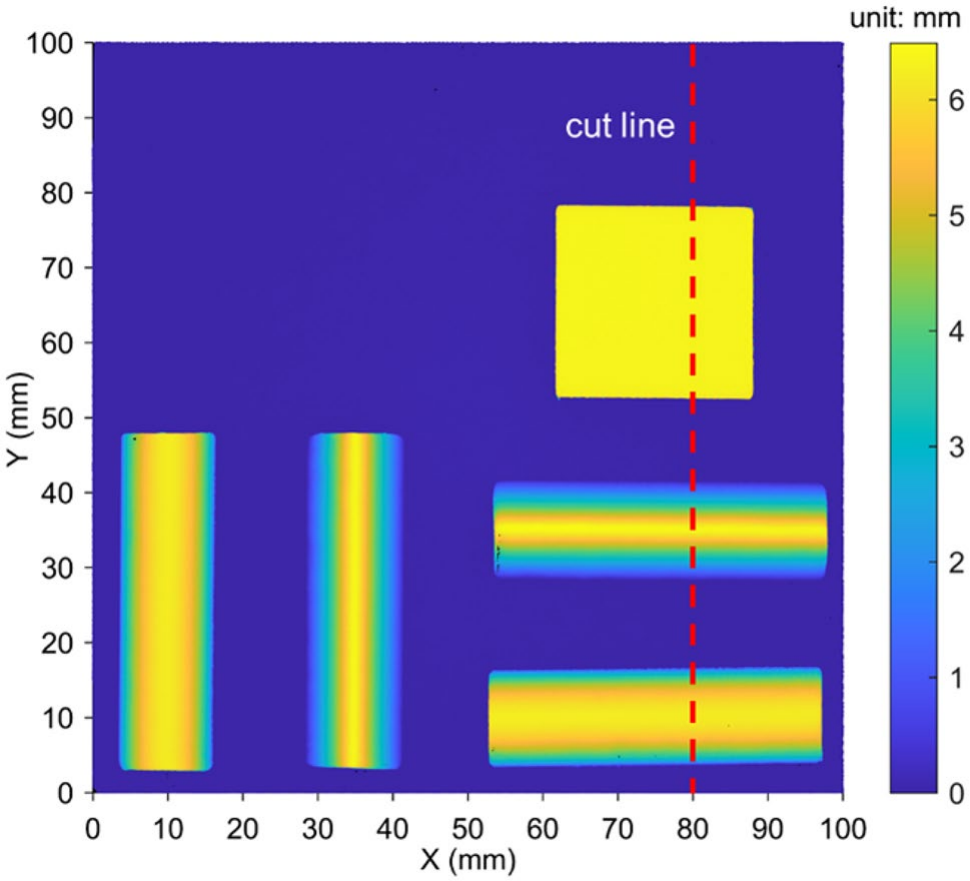
Laser scanning data.

As shown in Fig. 9, the laser scanning data has been transformed into the common coordinate system of 3D-DIC. To facilitate an intuitive comparison with the data obtained by 3D-DIC, the transformed height data of the tabletop, triangle, and cylinder are included through the wire cutting method.

**Fig. 10:**
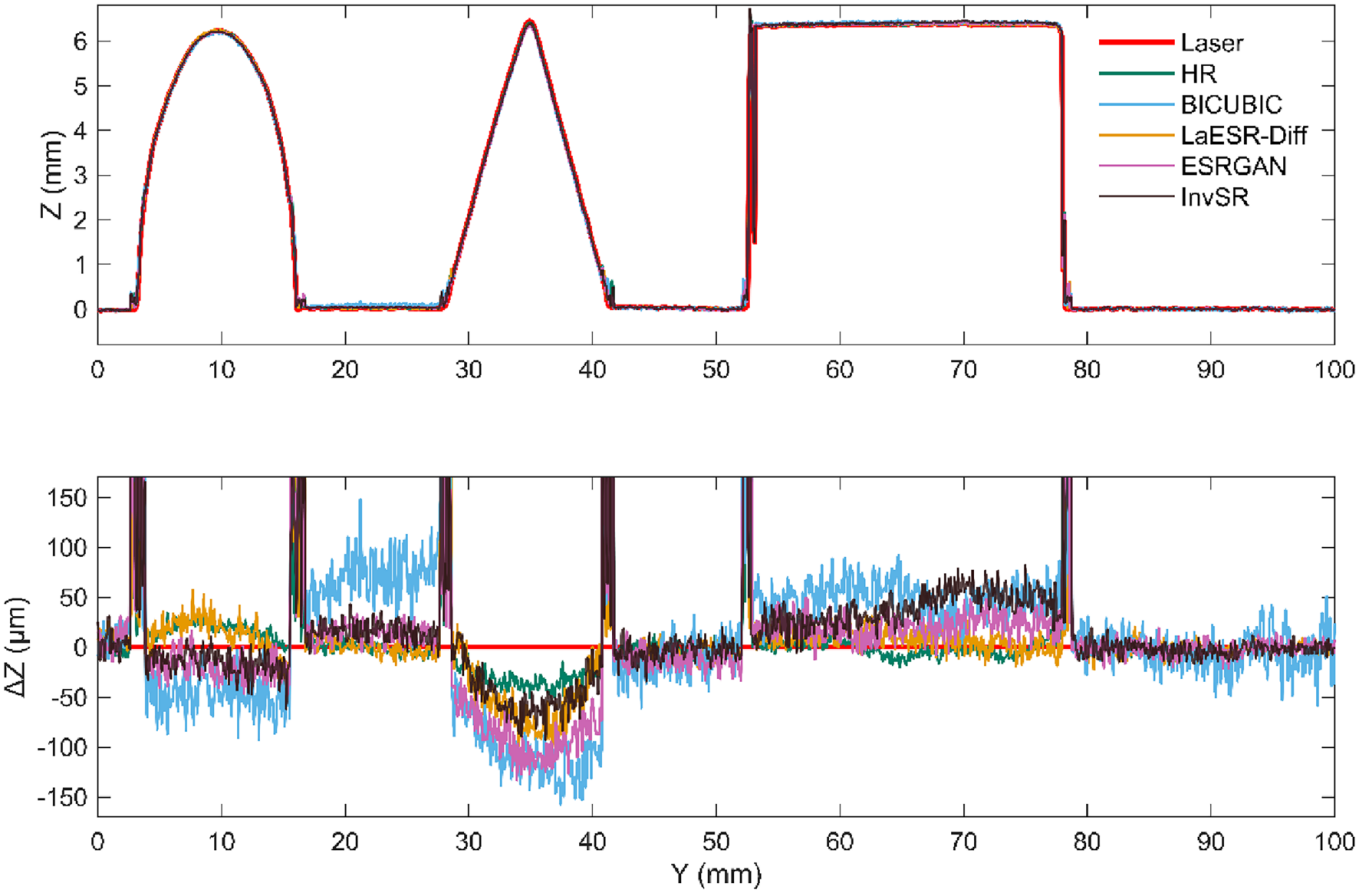
Comparison of 3D-DIC and Laser Scanning Results.

Fig. 10 shows the comparison between the results of 3D-DIC and the laser scanner after SR reconstruction of images using different methods under the condition of an image SR scale of × 4. The upper part of Fig. 10 is the result along the cutting line in the Z direction. It can be observed that at this display scale, the data of 3D-DIC is very close to that of the laser. Therefore, the difference $\Delta Z$ between the results of the laser and 3D-DIC in the Z direction is used for comparative analysis, and the calculation method of $\Delta Z$ is as follows:

28 $${\Delta Z=Z}_{3D-DIC}-{Z}_{Laser}$$

where ${Z}_{3D-DIC}$ represents the result of the Z-direction measurement by 3D-DIC, and ${Z}_{Laser}$ represents the result of the Z-direction measurement by the laser scanner. The lower part of Fig. 10 shows the result of $\Delta Z$. It can be seen that: 1) At the edges of the tabletop, triangle, and cylinder, the errors change sharply. This may be due to the interference of various factors on the measurement at the edges, such as occlusion, edge effects, etc.; 2) Except for the sharp changes at the edges, the errors in the flat part of the specimen and the tabletop area are relatively small, the errors in the cylinder area are slightly larger, and the errors in the triangle area are the largest. This indicates that the measurement error characteristics of different-shaped areas are different, which may be related to the geometric shape complexity of the areas; 3) After using the LaESR-Diff, ESRGAN and InvSR models for SR reconstruction of the images, compared with BICUBIC, they are closer to the original HR images, and the errors are significantly reduced, indicating that these models have better performance in image SR reconstruction for the 3D-DIC scenario.

**Fig. 11:**
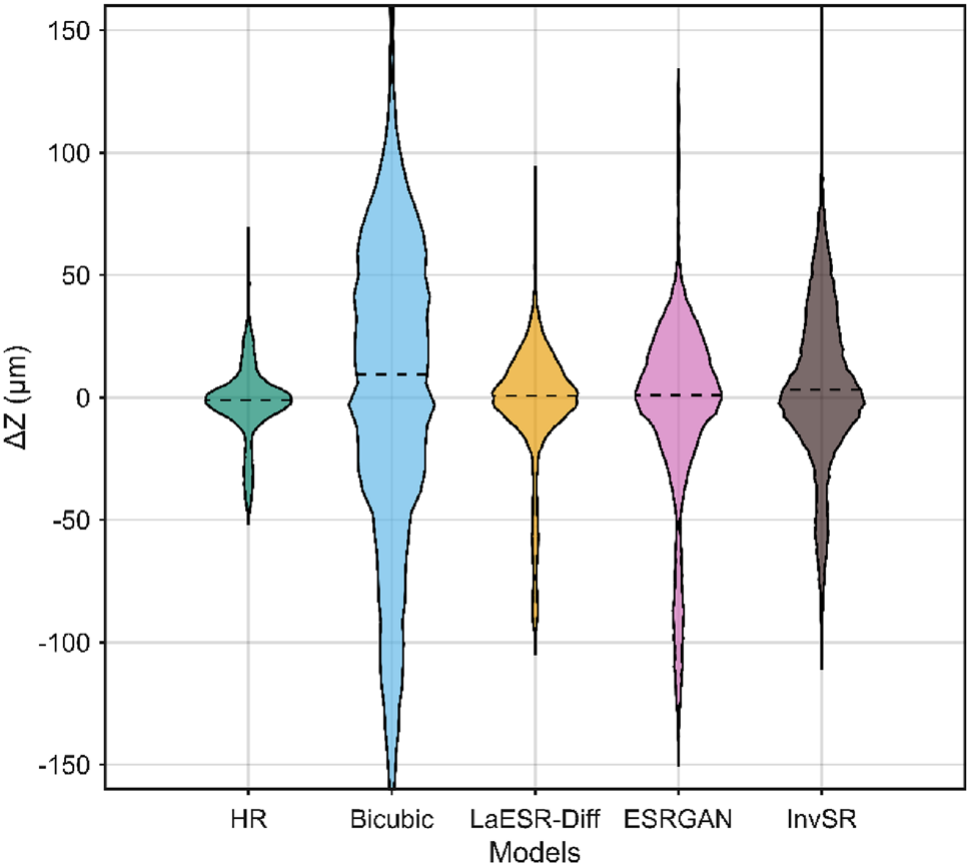
Error boxplots of different image SR models.

Fig. 11 is a box plot of the errors at all points on the specimen surface after excluding the sharp changes in measurement errors in the edge area in Fig. 10. It can be seen that the error distributions of different models are different. For example, the error distribution range of Bicubic may be relatively wide, indicating a large degree of error dispersion; while other models may perform better in terms of error concentration. The error dispersions of models such as LaESR-Diff, ESRGAN, and InvSR are relatively lower than that of the Bicubic model, and the reconstruction effects are relatively better, among which LaESR-Diff is superior to ESRGAN and InvSR.

Table 5 shows the results of the mean absolute error (i.e., the absolute average value of $\Delta Z$ of each model at different image SR scales. Among them, the boxed lines represent the best values in a single column, and the underlines represent the second-best values in a single column. The LaESR-Diff model obtained 2 best values and 1 s-best value. It is worth noting that at the × 8 scale, LaESR-Diff performed outstandingly, with its $\stackrel{-}{\left|\Delta Z\right|}$ reduced by 58.6% compared with BICUBIC, while the second-best InvSR was only reduced by 41.6% compared with BICUBIC, with a difference of 17.0%. In contrast, at the × 2 scale, the best-performing InvSR was reduced by 42.1% compared with BICUBIC, and the second-best LaESR-Diff was reduced by 39.9%, with a difference of only 2.2%. It can be seen that LaESR-Diff has a relatively obvious advantage in the task of 3D-DIC surface morphology measurement at higher image SR scales.

**Table 5:** The mean absolute error of each model under different image SR scales.

Model	$\left| {\Delta Z} \right|$(μm)		
	× 2	× 4	× 8
BICUBIC	40.1	58.2	173.6
ESRGAN^23^	24.7	30.4	122.9
InvSR	23.2	35.3	101.4
LaESR-Diff	24.1	25.2	71.9

**Fig. 12:**
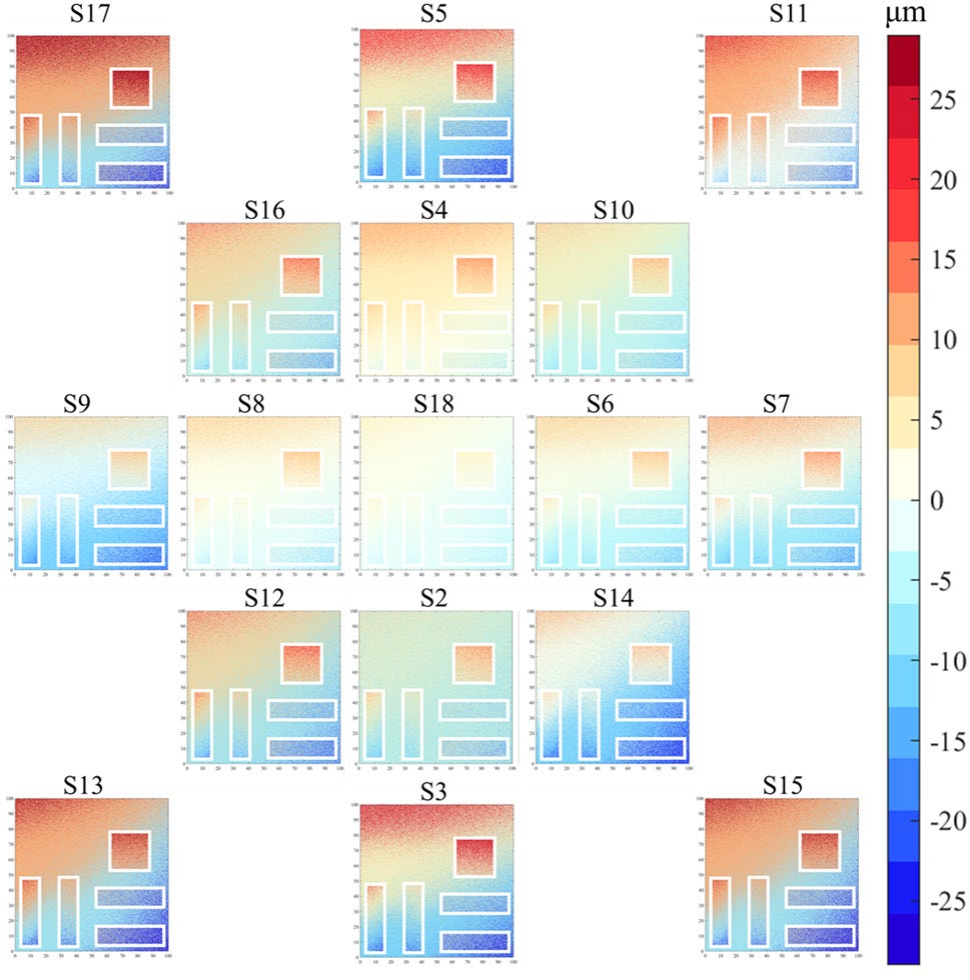
Distribution diagram of displacement measurement error after LaESR-Diff model reconstruction.

For the displacement measurement accuracy of the specimen, it is evaluated by the measurement error $\Delta A$ between 3D-DIC and the coordinate measuring machine:

29 $${\Delta A=A}_{3D-DIC}-{A}_{CMM}$$

where ${A}_{3D-DIC}$ is the displacement calculation result of 3D-DIC, and ${A}_{CMM}$ is the displacement measurement result of the coordinate measuring machine. Fig. 12 is an example of the displacement measurement error distribution diagram of the specimen, specifically the distribution diagram of the displacement measurement error $\Delta A$ after reconstruction by the LaESR-Diff model at the SR scale of × 4. Among them, S1—S18 correspond to the numbers of the specimen in different positions (see the lower part of Fig. 10 for details). Due to the influence of factors such as field-of-view occlusion during the image acquisition process, there are sharp changes in errors in the edge area, which are not the focus of this experiment. To more clearly show the displacement measurement error of the specimen, these areas with sharp error changes have been excluded, that is, the uncolored white areas in the figure. It can be observed from the figure that as the displacement in different directions increases, $\Delta A$ generally shows an increasing trend.

Table 6 shows the mean absolute error of displacement measurement (i.e., the absolute average value $\Delta A$ of the displacement measurement error ${\left|\Delta A\right|}$ of each model at different image SR scales. Among them, the boxed lines represent the best values in a single column, and the underlines represent the second-best values in a single column. The LaESR-Diff model achieved 3 best performances. At the × 8 scale, LaESR-Diff performed outstandingly, with its ${\left|\Delta A\right|}$ reduced by 67.2% compared with BICUBIC, while the second-best InvSR was only reduced by 54.3% compared with BICUBIC, with a difference of 12.9%. In contrast, at the × 4 scale, the difference in the reduction between the best-performing LaESR-Diff and the second-best InvSR compared with BICUBIC was 10.4%; at the × 2 scale, this difference was 7.0%. It can be seen that LaESR-Diff has an obvious advantage in the task of 3D-DIC displacement measurement at higher image SR scales, which is basically the same as the situation in the surface morphology measurement task.

**Table 6:** The mean absolute error of displacement measurement for each model under different image SR scales.

Model	${\left|\Delta A\right|}$(μm)		
	× 2	× 4	× 8
BICUBIC	45.9	70.4	278.4
ESRGAN^23^	29.8	32.4	162.2
InvSR	30.5	38.9	127.1
LaESR-Diff	27.3	31.6	81.4

**Fig. 13:**
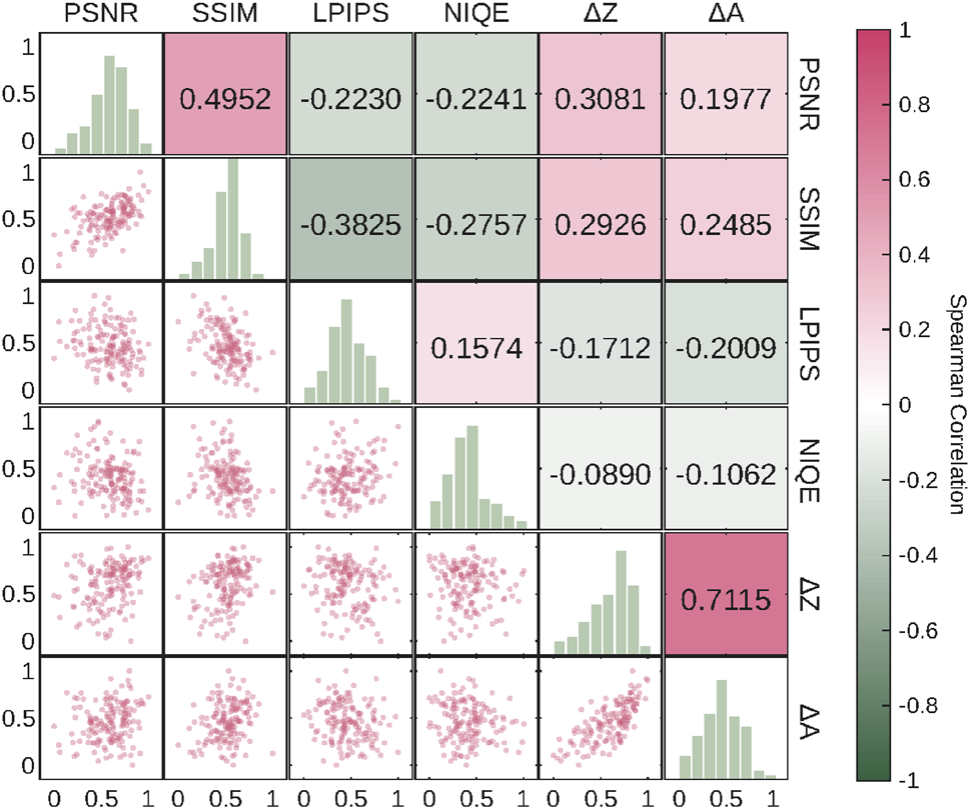
Multi metric correlation and distribution visualization matrix diagram.

Fig. 13 shows the visualization results of the multi-index evaluation matrix, including a scatter plot matrix, distribution histograms, and the Spearman rank correlation coefficients between every two of the 6 evaluation metrics. Spearman correlation is employed here to better capture the monotonic relationships between metrics, especially for perceptual and no-reference metrics that may not follow a strictly linear distribution. These metrics include 4 general image evaluation metrics (PSNR, SSIM, LPIPS, NIQE) and 2 evaluation metrics based on 3D-DIC measurement results ($\Delta Z$, $\Delta A$). To ensure data comparability, all metric values have been normalized to eliminate the influence of dimensions. In addition, to make the results more statistically significant, these metric results comprehensively cover all specimen images in this experiment, involving all super-resolution (SR) methods such as bicubic interpolation, LaESR-Diff, InvSR, ESRGAN, and data at all SR scales such as × 2, × 4, and × 8.

The Spearman correlation coefficient between PSNR and SSIM is 0.4952, showing a moderate positive correlation. This indicates that the two have a consistent ranking trend in measuring image quality; that is, images with higher pixel-level accuracy often also achieve higher structural similarity rankings. PSNR shows a weak negative monotonic correlation with LPIPS (-0.2230) and NIQE (-0.2241). In contrast, SSIM exhibits a relatively stronger negative correlation with LPIPS (-0.3825) and NIQE (-0.2757). This suggests that the rankings provided by SSIM are more aligned with perceptual quality and naturalness rankings than those of PSNR, likely because SSIM accounts for structural information that is more relevant to human visual perception and deep-feature-based metrics like LPIPS.

The correlations between the four general metrics and the two DIC-specific metrics ($\Delta Z$, $\Delta A$) remain generally weak. While PSNR and SSIM show slight positive correlations (ranging from 0.19 to 0.31), LPIPS and NIQE show even weaker negative correlations. This reinforces the conclusion that general-purpose image metrics struggle to effectively capture the monotonic impact of SR reconstruction on specific 3D-DIC measurement tasks. Notably, $\Delta Z$ and $\Delta A$ exhibit a strong positive Spearman correlation of 0.7115, indicating highly consistent performance rankings between these two task-specific metrics. Overall, the results confirm that in DIC scenarios, traditional metrics provide limited reference value, necessitating the use of metrics that directly evaluate the accuracy of 3D-DIC measurement results.

Table 7 compares the model complexity and computational efficiency. While ESRGAN exhibits the lowest complexity, it often falls short in restoring high-fidelity speckle patterns for large-scale SR. In contrast, while diffusion-based models like InvSR offer high performance, they are often criticized for their heavy computational burden (33.8 M parameters). Our proposed LaESR-Diff optimizes the diffusion framework, resulting in a more deployable model that significantly reduces FLOPs and runtime compared to InvSR while maintaining the robustness required for 3D-DIC tasks.

**Table 7:** Comparison of model complexity and computational efficiency.

Model	Model Complexity			
	Params (M)	FLOPs (G)	Training Time (h) / Memory (GB)	Inference Time (s) / Memory (GB)
ESRGAN	11.9	18.3	10.5 / 5.9	4.7 / 1.5
InvSR	33.8	45.8	28.0 / 7.8	15.1 / 2.9
LaESR-Diff	21.6	35.1	19.9 / 6.8	9.4 / 2.7

## Laboratory experiment

### Dataset source

**Fig. 14:**
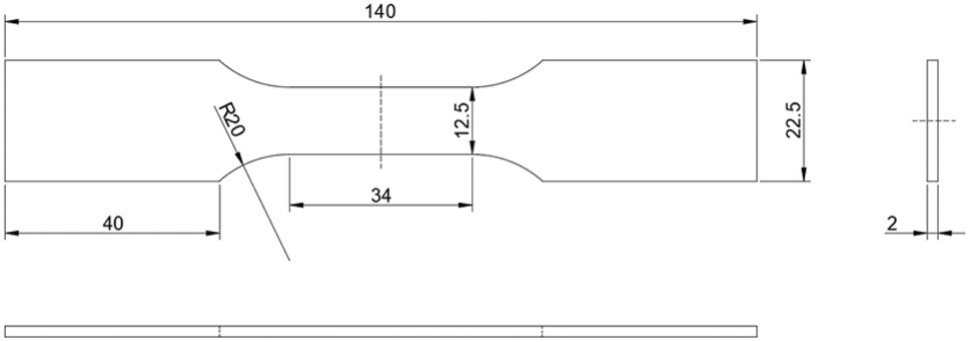
The tensile specimen dimensional parameters.

**Fig. 15:**
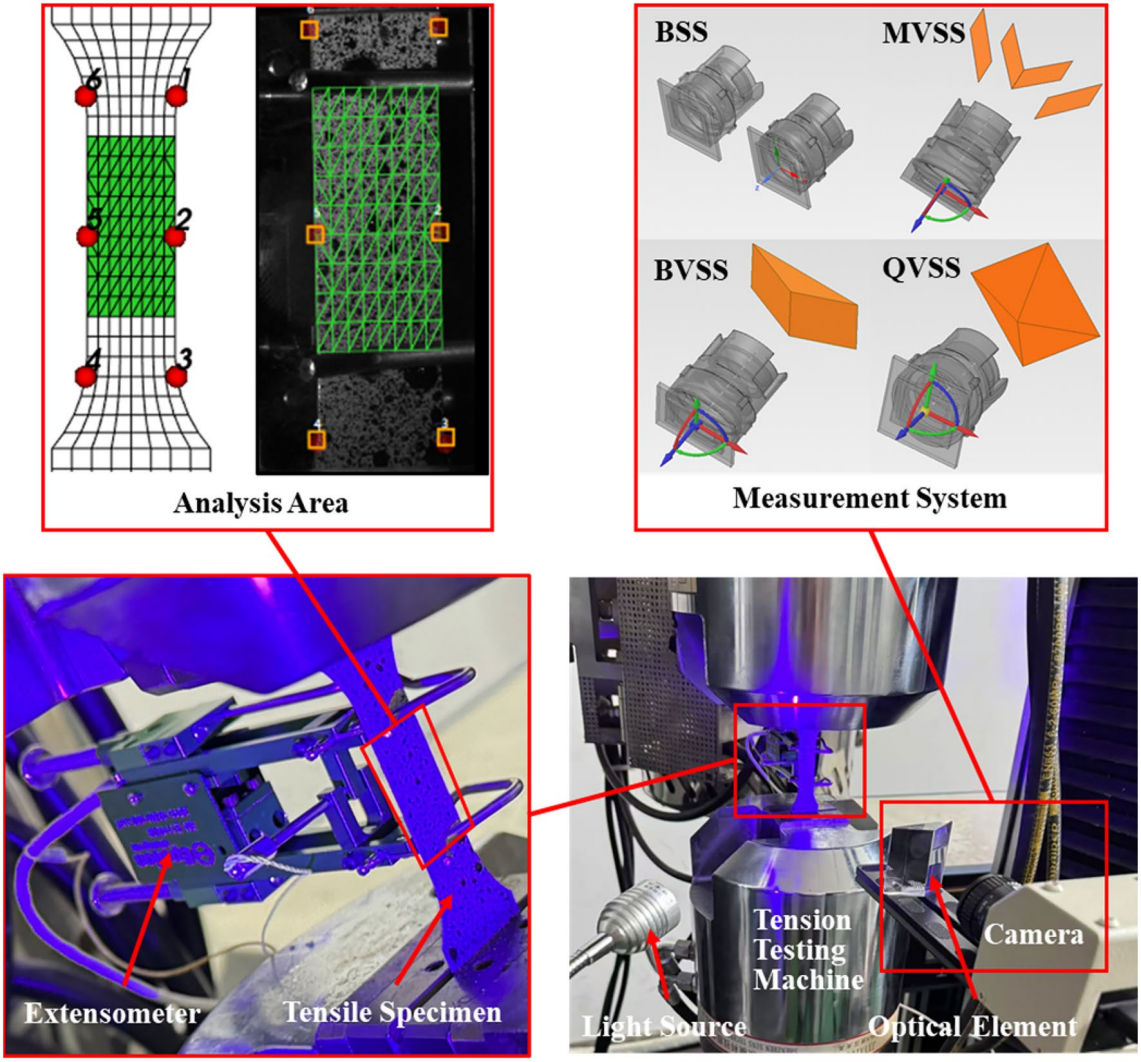
Schematic diagram of experimental site layout.

**Fig. 16:**
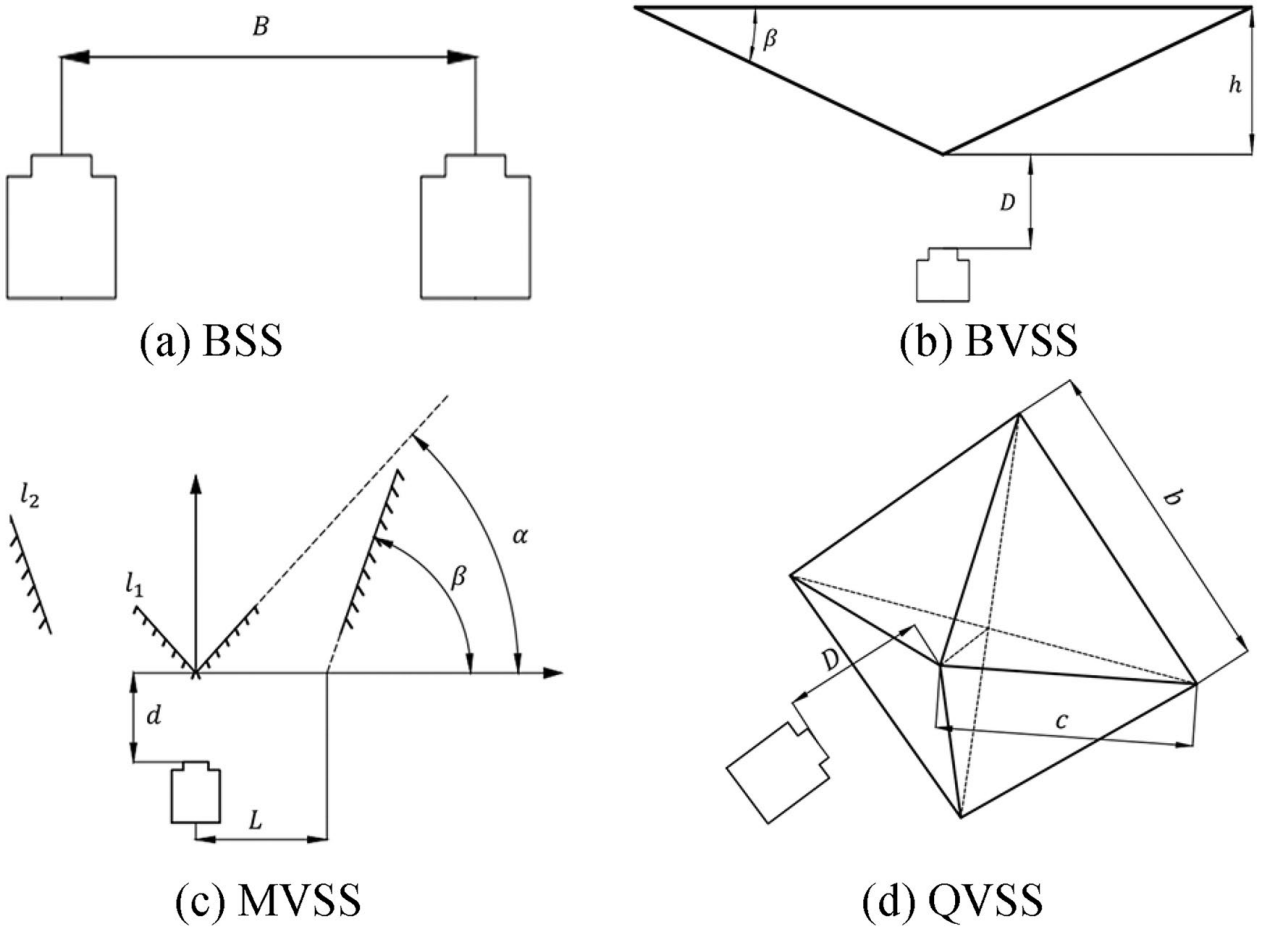
Schematic diagram of BSS, BVSS, MVSS, and QVSS structures.

**Fig. 17:**
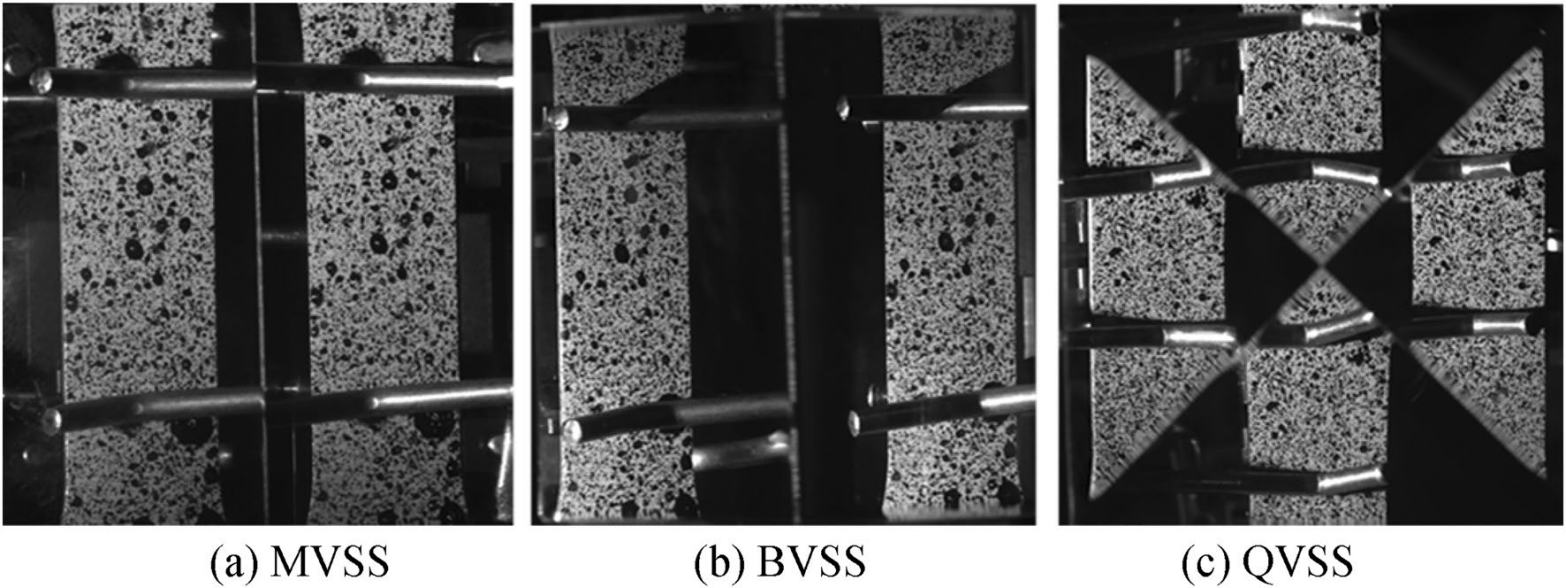
Example of image acquisition.

The tensile specimen in this experiment was made of steel, and local speckle spraying treatment was carried out on the specimen. Its dimensional parameters are shown in Fig. 14. The stretching speed of the tensile machine was 2 mm/min, and the stretching was stopped when the total displacement reached 0.333 mm, that is, the total stretching time was 10 s. The stretching process was recorded by a camera and an extensometer, and the measurement results of the extensometer were used as the reference values. Fig. 15 shows the experimental site layout. The arrangement of optical elements was divided into four cases: the basic stereo system (BSS) composed of two cameras; the mirror-based virtual stereo system (MVSS) composed of one camera and a plane mirror; the biprism-based virtual stereo system (BVSS) composed of one camera and a biprism; and the quadrangular-prism-based virtual stereo system (QVSS) composed of one camera and a quadrangular-prism. Fig. 16 shows the structural diagrams of the four stereo vision systems, and Table 8 lists the key geometric parameters. Fig. 17 shows examples of images collected by the single-camera stereo vision systems.

**Table 8:** The key geometric parameters of BSS, MVSS, BVSS, and QVSS.

**BSS**	
B (mm)	55
Objective distance (mm)	100
**MVSS**	
α (°)	45.5
β (°)	50.2
d (mm)	15.5
L (mm)	15.4
${l}_{1}$(mm)	20.5
${l}_{2}$(mm)	35.2
Objective distance (mm)	150
**BVSS**	
D (mm)	10
β (°)	22.5
h (mm)	8.5
Objective distance (mm)	160
**QVSS**	
D (mm)	10
b (mm)	25.6
c (mm)	20.2
Objective distance (mm)	200

### Results and analysis

**Fig. 18:**
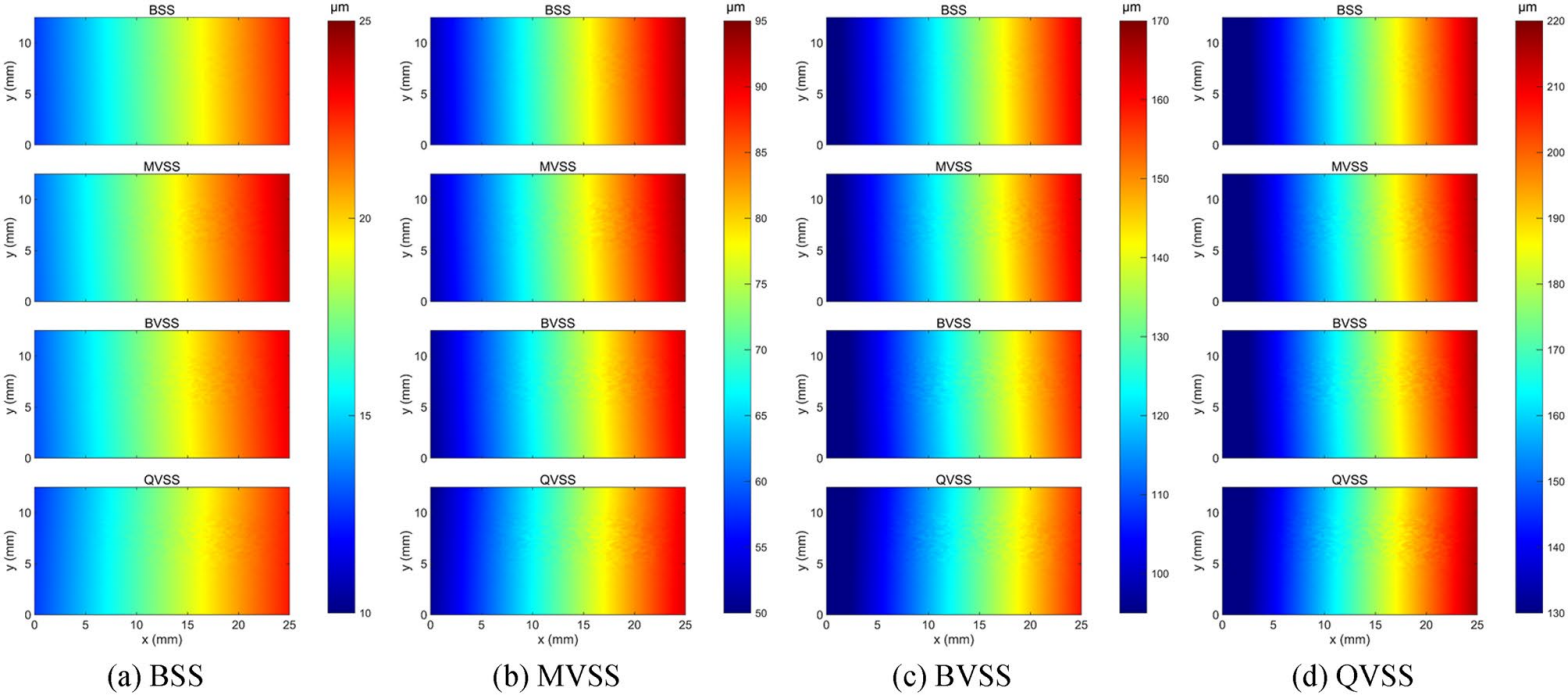
Full-field displacement maps.

**Fig. 19:**
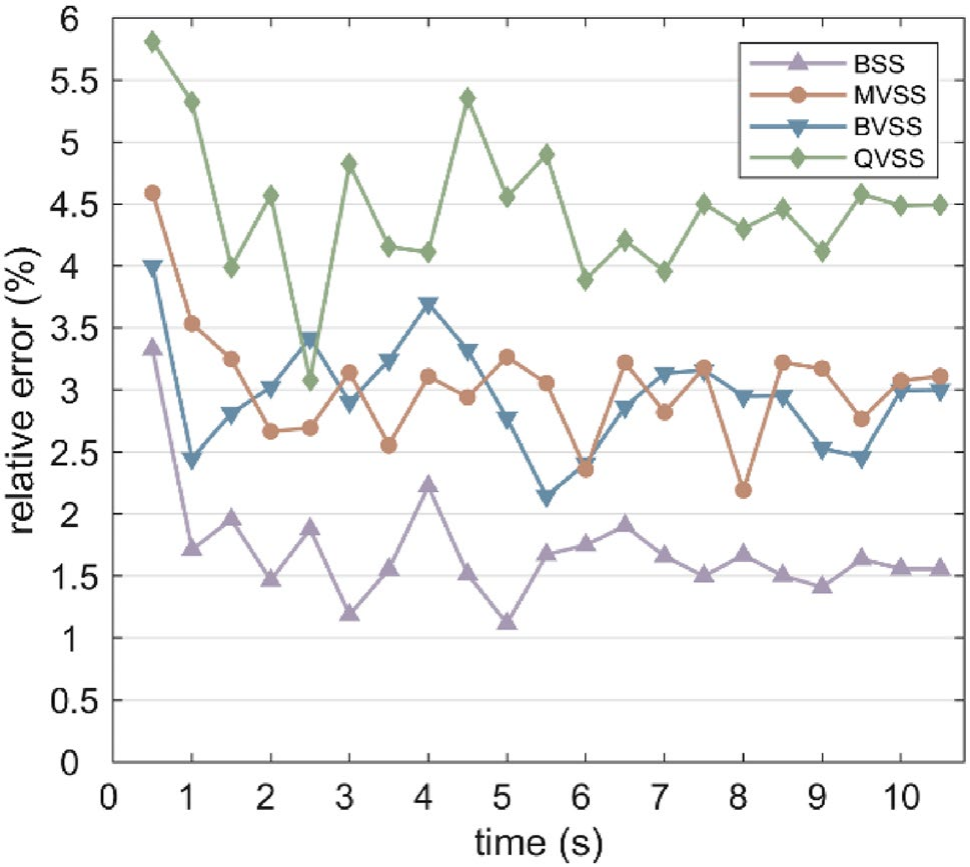
Relative errors of deformation measurement between 3D-DIC and the extensometer.

Fig. 18 shows the full-field displacement maps of the analysis area obtained by the 3D-DIC method at t = 1, 4, 7, and 10 s. From a visual perspective, the differences in the full-field displacement maps presented by different stereo measurement systems are not significant. To further explore the differences in full-field displacement measurement among different stereo measurement systems, more attention should be paid to quantitative metrics. Fig. 19 shows the relative errors of deformation measurement between 3D-DIC of the specimen and the extensometer. The results show that the overall relative error of BSS is the smallest, with an average relative error of 1.66%; the overall relative error of QVSS is the largest, with an average relative error of 4.25%; and the overall relative errors of MVSS and BVSS are relatively close, with average relative errors of 2.90% and 2.82%, respectively. This phenomenon can be attributed to the fact that compared with BSS, the image resolution of MVSS and BVSS is reduced by about 1/2, while that of the QVSS system is reduced by about 3/4, resulting in differences in relative errors. In addition, the relative errors of all measurement systems are large and fluctuate significantly in the initial stage, and gradually stabilize over time. This may be because in the initial stage of specimen stretching, the deformation is small, and the influence of minor disturbances in the environment and the inherent errors of the system on the measurement results is relatively prominent. As the stretching process continues and the specimen deformation increases, the measurement signal gradually strengthens, and the proportion of the influence of environmental disturbances and system errors decreases relatively, making the measurement results gradually stable.

**Fig. 20:**
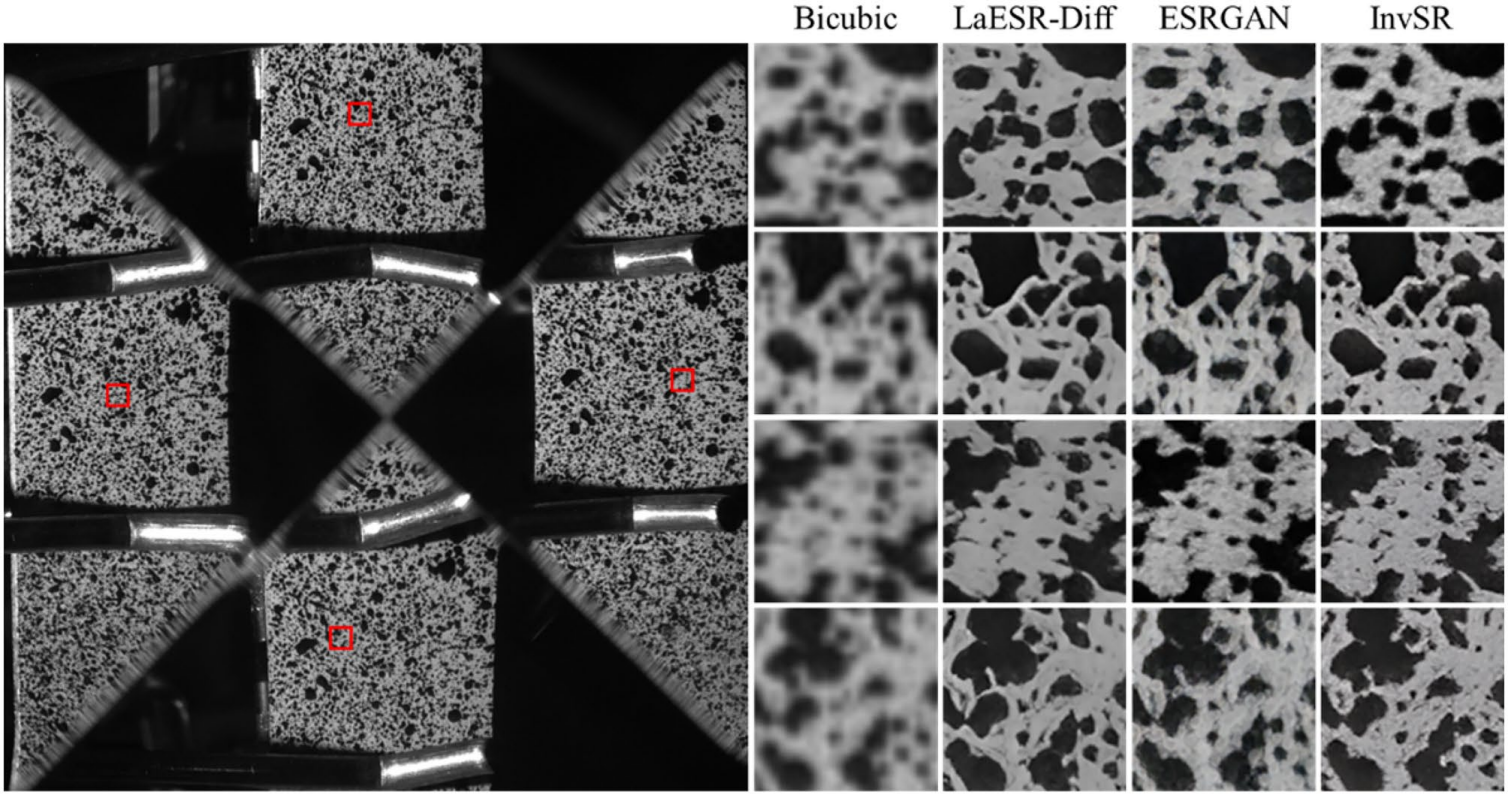
Comparison of SR results.

Fig. 20 presents an intuitive comparison of the local details of the specimen images after SR reconstruction by different methods. The image reconstructed by the Bicubic method is relatively blurry with indistinct texture features, showing poor visual effects. In contrast, the methods of ESRGAN, InvSR, and the LaESR-Diff method proposed in this study all improve the visual quality of the LR images to a certain extent. The image reconstructed by ESRGAN has enhanced textures, but there is still a certain degree of blurriness in the details. The image reconstructed by the InvSR method shows relatively rich details, but there are slight artifacts in some areas. In comparison, the LaESR-Diff method of this study has significant advantages in visual effects. The reconstructed image has a clear contour, is clean and sharp, without artifacts or blurriness.

**Fig. 21:**
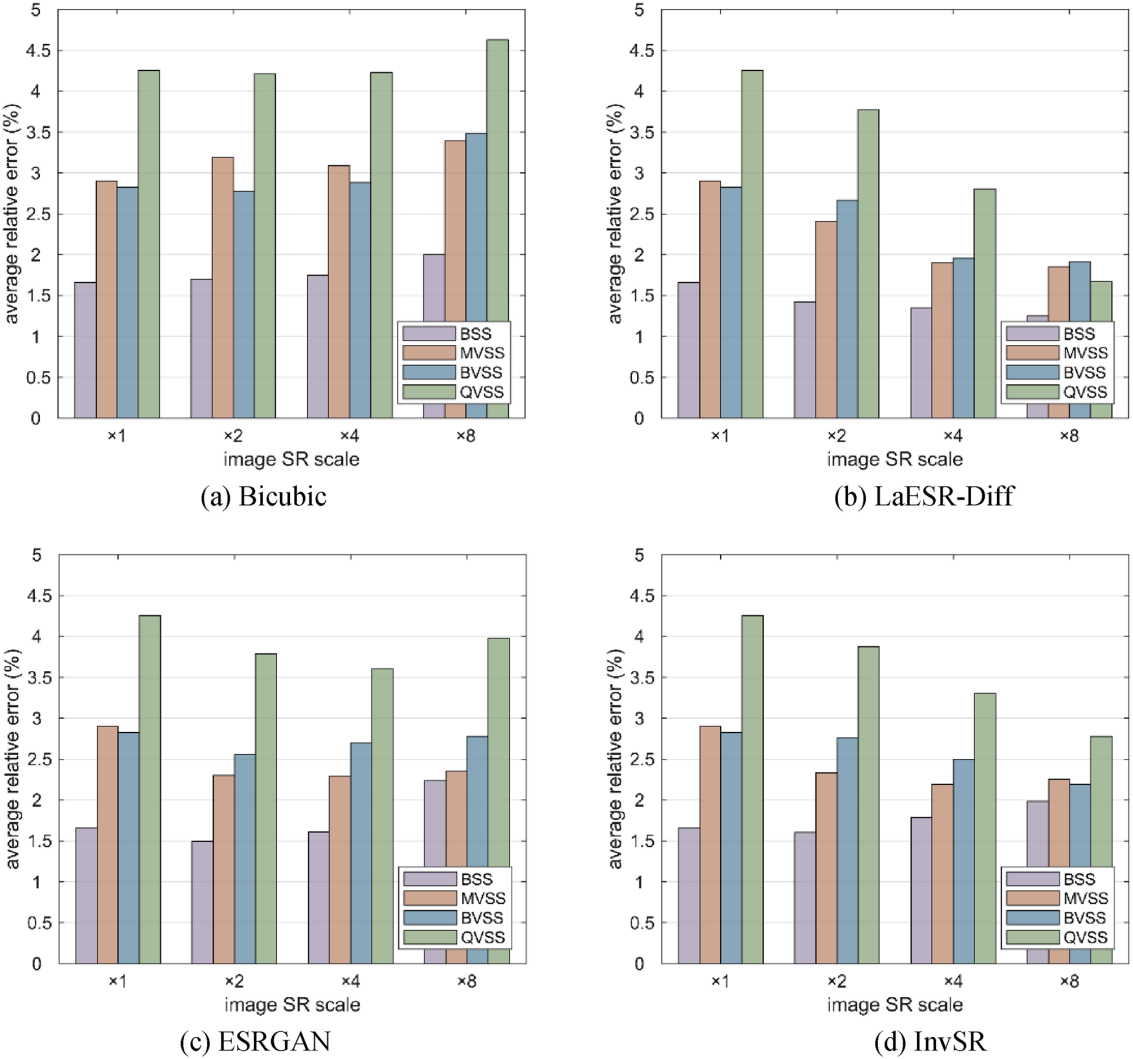
The average relative errors of deformation measurement between 3D-DIC and the extensometer after image SR reconstruction.

Fig. 21 shows the average relative errors of deformation measurement between 3D-DIC and the extensometer after image SR reconstruction. Fig. 21(a) presents the results using the Bicubic method. As the image SR magnification increases from × 1 to × 8, the average relative errors of each measurement system (BSS, MVSS, BVSS, QVSS) generally show an upward trend. Among them, the increase in the average relative error of QVSS is relatively prominent, reaching a relatively high level at × 8. Compared with other systems, the average relative error of BSS increases more gently, but the overall value also increases. The changes in the average relative errors of MVSS and BVSS are between those of QVSS and BSS. This indicates that when the Bicubic method is used for image SR reconstruction, as the magnification increases, it fails to effectively control the errors and even causes the errors of each measurement system to increase. Fig. 21(b) shows the results using the LaESR-Diff model. It can be observed that as the magnification increases, the average relative errors of each measurement system also decrease. The decrease in QVSS is still the most significant, the decrease in BSS is the least obvious, and the decreases in MVSS and BVSS are between the two. This shows that the LaESR-Diff model can also improve the accuracy of the stereo vision measurement system, and has a more significant improvement effect on the single-camera stereo vision measurement system. It is worth noting that at a magnification of × 8, the average relative error of QVSS is lower than that of MVSS and BVSS, and is equivalent to the performance of BSS at × 1. This indicates that by combining a well-performing image SR reconstruction model (such as LaESR-Diff), the multi-view single-camera stereo vision measurement system (QVSS) can effectively reduce the measurement error and, to a certain extent, compensate for the decrease in accuracy caused by image resolution loss. Fig. 21(c) shows the situation when the ESRGAN model is used. As the magnification gradually increases from × 1 to × 8, the trends of changes in the average relative errors of each measurement system are different. At low magnifications (× 1—× 2), the error changes of each system are relatively small; however, when the magnification further increases, especially at × 8, the average relative error of QVSS increases significantly, becoming the highest among several systems. The average relative error of BSS increases relatively slowly and remains at a relatively low level. The error increases of MVSS and BVSS are between the two. This shows that the ESRGAN model can maintain the accuracy of the measurement system to a certain extent, but as the magnification increases, its ability to control the errors of some systems (such as QVSS) weakens, and it is difficult to ensure the measurement accuracy at high magnifications. Fig. 21(d) presents the results of the InvSR model. As the magnification increases from × 1 to × 8, the average relative errors of MVSS and BVSS generally show a decreasing trend. For BSS, however, the error continues to increase. This indicates that the InvSR model has a negative effect on the accuracy improvement of BSS, and although its trend of improving the accuracy of QVSS is similar to that of the LaESR-Diff model, the improvement range is smaller. Comprehensive analysis shows that different models have significant differences in their impacts on the accuracy of each measurement system at different magnifications. The LaESR-Diff model has significant advantages at high magnifications, while other models have more or less accuracy problems at high magnifications; the BSS system is the least sensitive to image SR processing; and the QVSS system shows the most significant accuracy improvement after image SR reconstruction using the LaESR-Diff model.

## Discussion

The super-resolution diffusion model (LaESR-Diff) based on the optimized ESRGAN network and Laplace noise scheduling proposed in this study demonstrates significant advantages and application potential in 3D-DIC tasks. Introducing image super-resolution (SR) technology into 3D-DIC systems aims to improve measurement accuracy, especially for single-camera 3D-DIC systems with the problem of inherent image resolution loss. Although the single-camera stereo vision technology has the advantages of eliminating multi-camera synchronization errors, reducing hardware costs, and reducing the system volume, the resolution loss seriously restricts its measurement accuracy. The super-resolution reconstruction technology can effectively compensate for the resolution loss caused by optical design, maintain the advantages of the single-camera system, and compensate for the loss of measurement accuracy, providing new ideas for the development of 3D-DIC technology.

From the perspective of model performance, LaESR-Diff performs excellently in many aspects. In the public dataset experiment, compared with the traditional interpolation method Bicubic and other representative models ESRGAN and InvSR, LaESR-Diff has obvious advantages in PSNR and SSIM. At SR scales of × 2, × 4, and × 8, the PSNR values of LaESR-Diff reach 37.88, 35.09, and 26.13 respectively, and the SSIM values are 0.8964, 0.8522, and 0.7423 respectively. This indicates that the model performs outstandingly in the accurate prediction of image pixel values and the restoration of structural similarity. In 3D-DIC measurement tasks, LaESR-Diff has significant advantages in surface morphology measurement and displacement measurement tasks, especially at high image SR scales. At the × 8 scale, its average absolute error in surface morphology measurement is reduced by 58.6% compared with BICUBIC, and the average absolute error in displacement measurement is reduced by 67.2% compared with BICUBIC. This is mainly due to the targeted design of the LaESR-Diff model: on the one hand, the optimized ESRGAN model is used for conditional image input. By improving the Residual-in-Residual Dense Block (RRDB) unit, the network’s ability to extract the features of speckle images in 3D-DIC scenes is enhanced; the loss function is adjusted, and a loss term based on the Zero-mean Normalized Cross-Correlation (ZNCC) function is introduced, making the model’s optimization goal more consistent with the requirements of images in 3D-DIC scenes. On the other hand, the Laplace noise schedule is adopted, which effectively suppresses the smoothing effect of the diffusion process on fine textures, retains the local gradient features of the speckle pattern, and thus improves the quality of the reconstructed image and the 3D-DIC measurement accuracy.

In the tensile experiment, different stereo measurement systems show obvious differences in full-field displacement measurement. The overall relative error of BSS is the smallest, with an average relative error of 1.66%; the overall relative error of QVSS is the largest, with an average relative error of 4.25%; and the overall relative errors of MVSS and BVSS are relatively close, with average relative errors of 2.90% and 2.82%, respectively. This result is closely related to the degree of image resolution loss of each system. Compared with BSS, the image resolution of MVSS and BVSS is reduced by about 1/2, and that of the QVSS system is reduced by about 3/4, indicating the crucial influence of image resolution on 3D-DIC measurement accuracy. In the comparison of different image SR reconstruction models, the LaESR-Diff model performs excellently in improving the accuracy of the stereo vision measurement system, especially for the single-camera stereo vision measurement system (such as QVSS). At a magnification of × 8, after reconstruction using the LaESR-Diff model, the average relative error of QVSS is lower than that of MVSS and BVSS, and is equivalent to the performance of BSS at × 1, further demonstrating the effectiveness and superiority of the LaESR-Diff model in compensating for the resolution loss of the single-camera stereo vision system and improving the measurement accuracy.

The dual evaluation system constructed in this study is of great significance and innovative value. Traditional general image quality metrics (such as PSNR, SSIM, LPIPS, NIQE) have limitations in measuring the impact of image SR reconstruction on 3D-DIC measurement performance, and they have weak correlations with 3D-DIC measurement accuracy metrics (such as surface height reconstruction error, displacement measurement accuracy, deformation measurement accuracy). This study introduces 3D-DIC-specific measurement accuracy evaluation metrics. This evaluation strategy not only examines the visual quality of SR reconstruction but also focuses on verifying the actual gains of super-resolution processing on the final measurement accuracy, thus providing a more persuasive evaluation basis for the application of SR technology in experimental mechanics. This research idea breaks away from the traditional evaluation mode that only focuses on visual effects and is of great significance for promoting the paradigm shift of super-resolution technology from “visual enhancement” to “measurement enhancement”. While we primarily benchmark against Bicubic, ESRGAN, and InvSR, we acknowledge recent advancements in diffusion-based super-resolution, such as Image SR Via Iterative Refinement by^32^ and SinSR by^54^. These methods demonstrate impressive perceptual quality on natural images. However, our proposed LaESR-Diff is specifically optimized for the high-frequency speckle patterns inherent to 3D-DIC through the Laplacian noise schedule and ZNCC loss, which are not present in generic SOTA models.

However, this study still has certain limitations. At the experimental level, although the public dataset and tensile experiment were used for verification, the diversity of scenarios and samples is insufficient. It is necessary to increase experiments under complex working conditions to evaluate the generalization ability of the model. In terms of model evaluation, the computational complexity of the model has not been further evaluated, and the real-time performance of the algorithm has not been assessed. In terms of model optimization, the black-box nature of deep learning leads to high experimental costs for parameter optimization. In the future, interpretable models can be explored, and prior knowledge can be integrated to improve the adaptability and accuracy in complex scenarios.

## Conclusions

Aiming at the problem of decreased measurement accuracy caused by image resolution loss in single-camera three-dimensional digital image correlation (3D-DIC) systems, this study proposes a super-resolution diffusion model (LaESR-Diff) based on the optimized Enhanced Super-Resolution Generative Adversarial Network (ESRGAN) and Laplace noise scheduling, constructs a dual evaluation system suitable for 3D-DIC scenes, and systematically demonstrates the effectiveness and superiority of the proposed method through public datasets and laboratory tensile experiments. The main conclusions are as follows:

1. In the design of the LaESR-Diff model, the Residual-in-Residual Dense Block (RRDB) unit of ESRGAN is improved, a loss term based on the Zero-mean Normalized Cross-Correlation (ZNCC) function is introduced, and the Laplace noise schedule is adopted to replace the traditional linear or cosine scheduling. The aim is to make the model more accurately retain the local structural features of speckle images when generating conditional images and suppress the smoothing effect of the diffusion process on high-frequency textures.
2. In the public dataset experiment, the PSNR values of LaESR-Diff at × 2, × 4, and × 8 scales reach 37.88, 35.09, and 26.13 respectively, and the SSIM values are 0.8964, 0.8522, and 0.7423 respectively, which are significantly better than the traditional interpolation method (Bicubic) and the comparison models (ESRGAN, InvSR). In 3D-DIC measurement tasks, at the × 8 scale, the surface morphology measurement error is reduced by 58.6%, and the displacement measurement error is reduced by 67.2%, verifying the model’s ability to accurately restore image details in high-SR scale reconstruction and significantly improve the measurement accuracy.
3. The Pearson correlation coefficients between traditional general image quality metrics (such as PSNR, SSIM) and 3D-DIC measurement accuracy metrics (surface height error, displacement error) are all less than 0.3, indicating that they are difficult to effectively reflect the actual gains of super-resolution technology in measurement tasks. The dual evaluation system constructed in this study integrates general image quality metrics and 3D-DIC-specific metrics (such as surface height reconstruction error, displacement measurement accuracy), innovatively verifying the application effect of image SR reconstruction in 3D-DIC scenes from the two dimensions of “visual quality” and “measurement performance”, and providing a more targeted scientific basis for the evaluation of image SR technology in the field of experimental mechanics.
4. In the laboratory tensile experiment, for the single-camera systems based on plane mirrors (MVSS), biprisms (BVSS), and quadrangular pyramid prisms (QVSS), after being processed by the LaESR-Diff model, the full-field displacement measurement errors are significantly reduced. Among them, the average relative error of the QVSS system at the × 8 scale is reduced from 4.25% to 1.71%, approaching the 1.66% level of the dual-camera system (BSS), proving that super-resolution technology can effectively compensate for the accuracy defects of single-camera systems caused by resolution loss. Compared with other models, the LaESR-Diff model has a significantly better effect in improving the accuracy of single-camera systems at high magnifications than ESRGAN and InvSR, especially in multi-view single-camera systems (such as QVSS).

This study provides an effective image super-resolution algorithm-based solution for improving the accuracy of 3D-DIC systems, especially single-camera 3D-DIC. The research results show that super-resolution technology can effectively compensate for the measurement accuracy loss caused by resolution loss while maintaining the inherent advantages of single-camera systems. The related methods and ideas can also be extended to other image-based non-contact measurement fields, expanding the research ideas at the intersection of experimental mechanics and computer vision. Future research can be carried out from aspects such as expanding the diversity of experimental scenarios, optimizing the model algorithm architecture, and exploring model interpretability.

## Data Availability

The datasets generated and analyzed during the current study are available from the corresponding author on reasonable request.

## References

[1] Strungar, E. M., Yankin, A. S., Zubova, E. M., Babushkin, A. V. & Dushko, A. N. Experimental study of shear properties of 3D woven composite using digital image correlation and acoustic emission. *Acta Mech. Sin.* **36**, 448–459. 10.1007/s10409-019-00921-7 (2020).

[2] Luo, H., Yu, L. & Pan, B. Design and validation of a demand-oriented single-camera stereo-DIC system with a four-mirror adapter. *Measurement* **186**, 110083. 10.1016/j.measurement.2021.110083 (2021).

[3] Chouhan, K. & Chavda, J. T. A review on digital image correlation in experimental geotechnics. *Indian Geotech. J.*10.1007/s40098-023-00783-8 (2023).

[4] Yang, H. et al. Inverse identification of in-situ curing shrinkage using a method combining 3D digital image correlation and finite-element simulation. *Measurement* **223**, 113760. 10.1016/j.measurement.2023.113760 (2023).

[5] Yang, H. et al. Stress field identification using deep learning and three-dimensional digital image correlation. *Measurement* **244**, 116517. 10.1016/j.measurement.2024.116517 (2025).

[6] Yuan, Y. et al. Accurate displacement measurement via a self-adaptive digital image correlation method based on a weighted ZNSSD criterion. *Opt. Lasers Eng.* **52**, 75–85. 10.1016/j.optlaseng.2013.07.016 (2014).

[7] Pan, B. Digital image correlation for surface deformation measurement: Historical developments, recent advances and future goals. *Meas. Sci. Technol.* **29**, 082001. 10.1088/1361-6501/aac55b (2018).

[8] Pan, B., Yu, L. & Zhang, Q. Review of single-camera stereo-digital image correlation techniques for full-field 3D shape and deformation measurement. *Sci. China Technol. Sci.* **61**, 2–20. 10.1007/s11431-017-9090-x (2018).

[9] Pankow, M., Justusson, B. & Waas, A. M. Three-dimensional digital image correlation technique using single high-speed camera for measuring large out-of-plane displacements at high framing rates. *Appl. Opt.* **49**, 3418. 10.1364/AO.49.003418 (2010). 20539362 10.1364/AO.49.003418

[10] Genovese, K., Casaletto, L., Rayas, J. A., Flores, V. & Martinez, A. Stereo-Digital Image Correlation (DIC) measurements with a single camera using a biprism. *Opt. Lasers Eng.* **51**, 278–285. 10.1016/j.optlaseng.2012.10.001 (2013).

[11] Yang, B., Cen, X., Xie, L. & Yin, M. Automatic pose measurement of robotic drilling system based on zoom monocular vision. *Adv. Eng. Inform.* **65**, 103121. 10.1016/j.aei.2025.103121 (2025).

[12] Goshtasby, A. & Gruver, W. A. Design of a single-lens stereo camera system. *Pattern Recogn.* **26**, 923–937. 10.1016/0031-3203(93)90058-5 (1993).

[13] Mohammed, M. E., Shao, X. & He, X. Portable device for the local three-dimensional deformation measurement using a single camera. *Sci. China Technol. Sci.* **61**, 51–60. 10.1007/s11431-017-9078-0 (2018).

[14] Wu, F. et al. Curvelet coefficient prediction-based image super-resolution method for precision measurement. *Measurement* **222**, 113555. 10.1016/j.measurement.2023.113555 (2023).

[15] Liu, B., Yang, Y. & Chen, H. A lightweight convolutional neural network super-resolution method to improve the quality of target image in space measurement tasks. *Measurement* **242**, 116242. 10.1016/j.measurement.2024.116242 (2025).

[16] Chen, H., Li, H., Yao, C., Liu, G. & Wang, Z. Image super-resolution based on improved ESRGAN and its application in camera calibration. *Measurement* **242**, 115899. 10.1016/j.measurement.2024.115899 (2025).

[17] Ha, V. K. et al. Deep learning based single image super-resolution: A survey. *Int. J. Autom. Comput.* **16**, 413–426. 10.1007/s11633-019-1183-x (2019).

[18] Yu, M., Shi, J., Xue, C., Hao, X. & Yan, G. A review of single image super-resolution reconstruction based on deep learning. *Multimed. Tools Appl.*10.1007/s11042-023-17660-4 (2023).38283725

[19] Wang, X. et al. A review of image super-resolution approaches based on deep learning and applications in remote sensing. *Remote Sens.* **14**, 5423. 10.3390/rs14215423 (2022).

[20] Ye, S., Zhao, S., Hu, Y. & Xie, C. Single-image super-resolution challenges: A brief review. *Electronics* **12**, 2975. 10.3390/electronics12132975 (2023).

[21] C. Dong, C.C. Loy, K. He, X. Tang. Learning a Deep Convolutional Network for Image Super-Resolution. *In: European conference on computer vision*. 184–199 10.1007/978-3-319-10593-2_13 (2014).

[22] C. Ledig, L. Theis, F. Huszar, J. Caballero, A. Cunningham, A. Acosta, A. Aitken, A. Tejani, J. Totz, Z. Wang, W. Shi. Photo-Realistic Single Image Super-Resolution Using a Generative Adversarial Network. *In: 2017 IEEE Conference on Computer Vision and Pattern Recognition (CVPR), IEEE*. Honolulu, HI, pp. 105–114 10.1109/CVPR.2017.19. (2017).

[23] Wang, X. et al. ESRGAN: Enhanced super-resolution generative adversarial networks. In *Computer Vision – ECCV 2018 Workshops* (eds Leal-Taixé, L. & Roth, S.) 63–79 (Springer International Publishing, 2019). 10.1007/978-3-030-11021-5_5.

[24] W. Zhang, Y. Liu, C. Dong, Y. Qiao. RankSRGAN: Generative Adversarial Networks With Ranker for Image Super-Resolution. *In: 2019 IEEE/CVF International Conference on Computer Vision (ICCV), IEEE*. Seoul, Korea (South), pp. 3096–3105 10.1109/ICCV.2019.00319 (2019).

[25] I.J. Goodfellow, J. Pouget-Abadie, M. Mirza, B. Xu, D. Warde-Farley, S. Ozair, A. Courville, Y. Bengio. Generative Adversarial Nets. In: Advances in Neural Information Processing Systems, Curran Associates, Inc., 2014. https://proceedings.neurips.cc/paper_files/paper/2014/hash/f033ed80deb0234979a61f95710dbe25-Abstract.html (accessed May 28, 2025).

[26] R. Wu, T. Yang, L. Sun, Z. Zhang, S. Li, L. Zhang. SeeSR: Towards Semantics-Aware Real-World Image Super-Resolution. *In: 2024 IEEE/CVF Conference on Computer Vision and Pattern Recognition (CVPR), IEEE*. Seattle, WA, USA, pp. 25456–25467 10.1109/CVPR52733.2024.02405 (2024).

[27] Z. Yue, K. Liao, C.C. Loy. Arbitrary-steps Image Super-resolution via Diffusion Inversion. 10.48550/arXiv.2412.09013 (2025).

[28] Y. Song, J. Sohl-Dickstein, D.P. Kingma, A. Kumar, S. Ermon, B. Poole. Score-Based Generative Modeling through Stochastic Differential Equations. 10.48550/arXiv.2011.13456 (2021).

[29] J. Ho, A. Jain, P. Abbeel, Denoising Diffusion Probabilistic Models. In: Advances in Neural Information Processing Systems, Curran Associates, Inc., 2020: pp. 6840–6851. https://proceedings.neurips.cc/paper/2020/hash/4c5bcfec8584af0d967f1ab10179ca4b-Abstract.html (accessed May 28, 2025).

[30] R. Rombach, A. Blattmann, D. Lorenz, P. Esser, B. Ommer. High-Resolution Image Synthesis with Latent Diffusion Models. *In: 2022 IEEE/CVF Conference on Computer Vision and Pattern Recognition (CVPR), IEEE*. New Orleans, LA, USA, pp. 10674–10685 10.1109/CVPR52688.2022.01042 (2022).

[31] Moser, B. B. et al. Diffusion models, image super-resolution, and everything: A survey. *IEEE Trans. Neural Netw. Learn. Syst.* **36**, 1–21. 10.1109/TNNLS.2024.3476671 (2024). 10.1109/TNNLS.2024.3476671 39471123

[32] Saharia, C. et al. Image super-resolution via iterative refinement. *IEEE Trans. Pattern Anal. Mach. Intell.* **45**, 1–14. 10.1109/TPAMI.2022.3204461 (2022). 36094974 10.1109/TPAMI.2022.3204461

[33] Liu, Y. et al. SR-M−GAN: A generative model for high-fidelity stress fields prediction of the composite bolted joints. *Adv. Eng. Inform.* **61**, 102537. 10.1016/j.aei.2024.102537 (2024).

[34] Chen, L., Meng, K., Zhang, H., Zhou, J. & Lou, P. SR-FABNet: Super-resolution branch guided Fourier attention detection network for efficient optical inspection of nanoscale wafer defects. *Adv. Eng. Inform.* **65**, 103200. 10.1016/j.aei.2025.103200 (2025).

[35] Pan, B., Yu, L. & Wu, D. High-accuracy 2D digital image correlation measurements with bilateral telecentric lenses: Error analysis and experimental verification. *Exp. Mech.* **53**, 1719–1733. 10.1007/s11340-013-9774-x (2013).

[36] H. Wang, X. Wang, W. Sun, Z. Wang, G. Li. Application of super-resolution camera calibration method in DIC measurements. In: K. Shimizu, Y. Ma (Eds.), *Fourth International Conference on Advanced Optics and Photonics Research in Engineering (AOPR 2024)*. SPIE, Wuhan, China, p. 18 10.1117/12.3045173 (2024).

[37] K. Ezhova, D.H. Nguyen, D. Fedorenko. Development of a measuring system based on the principles of stereo vision. In: B. Bodermann, K. Frenner, R.M. Silver (Eds.), Modeling Aspects in Optical Metrology VII, SPIE, Munich, Germany, p. 43 10.1117/12.2525630. (2019).

[38] Chen, H., Liu, G. & Wang, Z. A stereovision-based efficient measurement approach for surface flatness of concrete members. *Structures* **63**, 106374. 10.1016/j.istruc.2024.106374 (2024).

[39] Long, L. et al. Binocular vision-based pose monitoring technique for assembly alignment of precast concrete components. *Adv. Eng. Inform.* **65**, 103205. 10.1016/j.aei.2025.103205 (2025).

[40] Pan, B., Xie, H. & Wang, Z. Equivalence of digital image correlation criteria for pattern matching. *Appl. Opt.* **49**, 5501. 10.1364/AO.49.005501 (2010). 20885489 10.1364/AO.49.005501

[41] Jailin, C. Full field modal measurement with a single standard camera. *Opt. Lasers Eng.* **107**, 265–272. 10.1016/j.optlaseng.2018.03.031 (2018).

[42] M. Inaba, T. Hara, H. Inoue. A stereo viewer based on a single camera with view-control mechanisms. *In: Proceedings of 1993 IEEE/RSJ International Conference on Intelligent Robots and Systems (IROS ‘93), IEEE*. Yokohama, Japan, pp. 1857–1865 10.1109/IROS.1993.583888. (1993).

[43] Y. Zhang, Y. Tian, Y. Kong, B. Zhong, Y. Fu. Residual Dense Network for Image Super-Resolution, in: 2018: pp. 2472–2481. https://openaccess.thecvf.com/content_cvpr_2018/html/Zhang_Residual_Dense_Network_CVPR_2018_paper.html (accessed February 27, 2024).

[44] C. Wang, Z. Li, J. Shi. Lightweight image super-resolution with adaptive weighted learning network. 10.48550/arXiv.1904.02358 (2019).

[45] E. Hoogeboom, J. Heek, T. Salimans. Simple diffusion: End-to-end diffusion for high resolution images, in: Proceedings of the 40th International Conference on Machine Learning, PMLR, 2023: pp. 13213–13232. https://proceedings.mlr.press/v202/hoogeboom23a.html (accessed May 28, 2025).

[46] Wang, J., Yue, Z., Zhou, S., Chan, K. C. K. & Loy, C. C. Exploiting diffusion prior for real-world image super-resolution. *Int. J. Comput. Vis.* **132**, 5929–5949. 10.1007/s11263-024-02168-7 (2024).

[47] C. Wang, Z. Hao, Y. Tang, J. Guo, Y. Yang, K. Han, Y. Wang. SAM-DiffSR: Structure-Modulated Diffusion Model for Image Super-Resolution, (2024). http://arxiv.org/abs/2402.17133 (accessed August 26, 2024).

[48] T. Hang, S. Gu. Improved Noise Schedule for Diffusion Training, (2024). http://arxiv.org/abs/2407.03297 (accessed September 2, 2024).

[49] Wang, Z., Bovik, A. C., Sheikh, H. R. & Simoncelli, E. P. Image quality assessment: From error visibility to structural similarity. *IEEE Trans. Image Process.* **13**, 600–612. 10.1109/TIP.2003.819861 (2004). 15376593 10.1109/tip.2003.819861

[50] R. Zhang, P. Isola, A.A. Efros, E. Shechtman, O. Wang. The Unreasonable Effectiveness of Deep Features as a Perceptual Metric. *In: 2018 IEEE/CVF Conference on Computer Vision and Pattern Recognition, IEEE*. Salt Lake City, UT, pp. 586–595 10.1109/CVPR.2018.00068. (2018).

[51] Mittal, A., Soundararajan, R. & Bovik, A. C. Making a “completely blind” image quality analyzer. *IEEE Signal Process. Lett.* **20**, 209–212. 10.1109/LSP.2012.2227726 (2013).

[52] Ahmad, W. Stereo-DIC challenge 1.0 – Rigid body motion of a complex shape. *Exp. Mech.*10.1007/s11340-024-01077-7 (2024).

[53] Ahmad, W. et al. Stereo-DIC challenge 1.0 – Rigid body motion of a complex shape. *Exp. Mech.* **64**, 1073–1106. 10.1007/s11340-024-01077-7 (2024).

[54] Y. Wang, W. Yang, X. Chen, Y. Wang, L. Guo, L.-P. Chau, Z. Liu, Y. Qiao, A.C. Kot, B. Wen. SinSR: Diffusion-Based Image Super-Resolution in a Single Step. *In: 2024 IEEE/CVF Conference on Computer Vision and Pattern Recognition (CVPR), IEEE*. Seattle, WA, USA, pp. 25796–25805 10.1109/CVPR52733.2024.02437. (2024).

